# A Persistent Hotspot of *Schistosoma mansoni* Infection in a Five-Year Randomized Trial of Praziquantel Preventative Chemotherapy Strategies

**DOI:** 10.1093/infdis/jix496

**Published:** 2017-09-16

**Authors:** Ryan E Wiegand, Pauline N M Mwinzi, Susan P Montgomery, YuYen L Chan, Kennedy Andiego, Martin Omedo, Geoffrey Muchiri, Michael O Ogutu, Fredrick Rawago, Maurice R Odiere, Diana M S Karanja, W Evan Secor

**Affiliations:** 1 Division of Parasitic Diseases and Malaria, Centers for Disease Control and Prevention, Atlanta, Georgia; 2 Center for Global Health Research, Kenya Medical Research Institute, Kisumu; 3 Emory University, Atlanta, Georgia

**Keywords:** mass drug administration, persistent hotspot, schistosomiasis, spatial clusters

## Abstract

**Background:**

Persistent hotspots have been described after mass drug administration (MDA) for the control of schistosomiasis, but they have not been studied during the course of a multiyear MDA program.

**Methods:**

In data from a 5-year study of school-based and village-wide preventive chemotherapy strategies for *Schistosoma mansoni*, spatial scan statistics were used to find infection hotspots in 3 populations: 5- to 8-year-olds, 9- to 12-year-olds, and adults. Negative binomial regression was used to analyze changes from baseline, and receiver operating characteristic analyses were used to predict which villages would reach prevalence and intensity endpoints.

**Results:**

We identified a persistent hotspot, not associated with study arm, where *S. mansoni* infection prevalence and intensity did not decrease as much as in villages outside the hotspot. Significant differences from baseline were realized after 1 year of MDA: we did not identify factors that moderated this relationship. Villages meeting specified endpoints at year 5 were predicted from prior year data with moderately high sensitivity and specificity.

**Conclusions:**

The MDA strategies were less effective at reducing prevalence and intensity in the hotspot compared with other villages. Villages that reached year 5 endpoints could be detected earlier, which may provide the opportunity to amend intervention strategies.

## Teaser

During a trial of preventive chemotherapy strategies, a hotspot of *Schistosoma mansoni* infection was found. Significant differences between hotspot and nonhotspot villages were found after 1 year. Villages meeting endpoints at year 5 were predicted from prior data with accuracy.

Schistosomiasis is a parasitic disease caused by members of the *Schistosoma* species, can be manifest in intestinal or urogenital forms, and is considered a public health problem in endemic areas. Human infection is acquired by contact with freshwater bodies containing schistosome cercariae that penetrate the skin. Morbidity results from eggs produced by adult female parasites that become lodged in host tissues [1].

Different strategies exist for the control of schistosomiasis including preventive chemotherapy, snail control with molluscicides or by other means, improvements to sanitation, greater access to safe water, and health education for behavior change [1]. Of these, mass drug administration (MDA) with praziquantel is the primary approach [2, 3]. An estimated 240 million people had schistosomiasis in 2010 [4], and more than 66.5 million people received preventive chemotherapy in 2015 in 52 endemic countries [5]. For 40 mg/kg dosages of praziquantel, a recent meta-analysis estimated cure rates of 77.1% and 76.7% and egg reduction rates of 94.1% and 86.3% for *Schistosoma haematobium* and *Schistosoma mansoni* infections, respectively [6]. Praziquantel remains inexpensive and is considered safe [7]. For these reasons, the World Health Organization (WHO) calls for the use of preventive chemotherapy for schistosomiasis annually, biennially, or twice during primary school depending on a village’s risk category [8].

Mass drug administration with praziquantel has led to reductions in both urogenital and intestinal schistosomiasis. However, some locations have maintained high levels of infection prevalence and intensity despite MDA. After more than 20 years of MDA in the Nile Delta, certain villages possessed high *S. mansoni* infection prevalence [3]. In western Côte d’Ivoire, an overall reduction in *S. mansoni* infection prevalence and intensity was achieved 1 year after a single, school-based MDA, yet 10.0% of schools saw an increase in *S. mansoni* infection prevalence by 25% or more [9]. In a cross-sectional study of 22 villages in the Philippines, 75.6% of participants reported being treated in the last 2 years, yet 13 of those 22 villages had a prevalence above 25% [10]. The causes of poor MDA efficacy could include reduced praziquantel effectiveness [11, 12], environmental factors [13], water contact [14], or a lack of participation [15].

We evaluated differences in MDA impact among 150 villages participating in a randomized trial of multiyear strategies for preventative chemotherapy in western Kenya. To our knowledge, this is the first attempt to search for a hotspot of schistosomiasis prevalence and intensity in a multiyear preventive chemotherapy trial. In Côte d’Ivoire, after 1 year of MDA during a similar cluster-randomized trial, maps suggested geographic clustering of villages, although no spatial clustering analyses were performed [9]. A study in Virgem das Graças, Brazil explored spatial clustering pre- and post-MDA but only for a single follow-up year [16]. Many examples of spatial clusters of schistosomiasis infection have been found, although these are from cross-sectional studies [17, 18] or surveillance data [19, 20].

Our analyses address 4 objectives. First, we evaluated the existence of hotspots in each year of the study in 3 age groups (5- to 8-year-olds, 9- to 12-year-olds, and adults). Our definition for a hotspot in these analyses was a spatial cluster of villages where there was significantly more schistosome infection than in those outside the cluster. Second, we estimated whether villages in year-1 hotspots experienced different changes from baseline of prevalence and infection intensity compared with nonhotspot villages. Third, we aimed to determine whether study arm, village characteristics, and environmental covariates moderated the changes from baseline. Fourth, we classified villages who fell below a target threshold at year 5 and determined whether data from prior years could reliably predict this classification.

## METHODS

### Study Design

The background for this project and study methods for this trial have been described in greater detail elsewhere [21–23]. In brief, a total of 150 villages near the eastern part of Lake Victoria in Kenya participated in this 5-year study. Villages were randomized to 1 of 6 treatment strategies (25 per arm), which involved a combination of school-based treatment and community-wide treatment (Supplementary Figure S1). Three study arms received annual MDA (4 treatments), and the remaining arms received MDA twice over 4 years. A repeated, cross-sectional sampling plan was used, meaning a different random sample was collected each year. Adults and first-year students were sampled in years 1, 3, and 5, had a sampling target of 50 participants per village, and provided 1 stool. In contrast, 9- to 12-year-olds were sampled every year, had a sampling target of 100 participants, and provided 3 stools. All stools were assessed in duplicate by the Kato-Katz technique for eggs of *S. mansoni*. In years 1–4, stool collection was followed by MDA administered in accordance with the villages’ treatment strategy. Year 5 was assessment only, although participants presenting with infection were treated. Villages not scheduled for MDA were not assessed for schistosome infection. Administration of praziquantel was a single dose of 40 mg/kg using the WHO-recommended dose pole [24].

### Data Collection and Analysis

Individual-level data were collected using barcoded master lists, entered into smart phones via EpiCollect software, and then transmitted to a central server at Imperial College (London, UK). Counts of schistosome eggs were averaged across all slides and converted to eggs per gram (EPG). Outcomes at the village level are used in these analyses including the following: prevalence, the percentage of participants with EPG >0; mean intensity, the mean EPG of all participants; median intensity, the median EPG of all participants; and high-intensity prevalence, the percentage of participants with EPG ≥400.

A survey including questions regarding sanitation, concurrent health interventions, and other information was administered to key informants from each village. Geographic coordinates for primary schools were collected with global positioning units (Trimble Navigation Ltd, Sunnyvale, CA), verified, and, if necessary, cleaned. Based on analyses of remote sensing data from a survey in 13- and 14-year-olds to determine village eligibility [25], we also downloaded available normalized difference vegetation index (NDVI) [26], land surface temperature [27] and rainfall [28] data from the time of the trial. Yearly averages of the remote sensing data at the school location and the closest point on the shoreline were created and then split into quartiles for analyses. All spatial data were projected to Universal Transverse Mercator zone 36S. Tests of differences over time in the distribution of prevalence and mean intensity were performed with the Kruskal-Wallis test.

#### Hotspots

Hotspots of infection were determined with the discrete-time Poisson spatial scan statistic [29] using prevalence and high-intensity prevalence and the normal variant [30] for mean and median intensity in SaTScan (version 9.4.2; Martin Kulldorff, Boston, MA). Each year and age cohort were run independently, and hotspots could be no larger than half of the sample in that age cohort and year. Maps were created using ArcGIS (version 10.3.1; ESRI, Redlands, CA). Primary clusters were included on each map as well as secondary clusters in locations where primary clusters existed in other years or cohorts. From this point forward, the clusters from SaTScan will be referred to as hotspots.

#### Changes From Baseline

Hotspots from year 1 were then used to estimate changes in prevalence and intensity from year 1 between villages inside and outside hotspots at subsequent years. Negative binomial models with generalized estimating equations [31] and an exchangeable correlation structure in SAS Software (version 9.3; SAS Institute, Inc., Cary, NC) were used to fit longitudinal models to prevalence and intensity data. Results are reported as a ratio of the change in prevalence from year 1 for mean of the villages inside the cluster compared with the villages outside the cluster (ie, a difference of differences). The distance to Lake Victoria was included as a covariate in all models, which removed the majority of the spatial variation, similar to analyses of the eligibility data [25].

To investigate the role of potential confounders, we repeated the analysis after matching villages within hotspot to villages outside the hotspot. Villages were matched on study arm, distance to Lake Victoria within 1 km, and baseline prevalence within 10%.

#### Moderators

Potential moderators were assessed based on the degree to which they affected the relationship between hotspot membership and prevalence or intensity. Selection of 2-way and 3-way interactions between year, hotspot membership, and each covariate was performed with the LASSO variable selection procedure [32]. Interaction terms that did not shrink to zero were considered as moderators. Because there were a limited number of villages in infection hotspots, we only included a single moderator at a time. Negative binomial regression [33] was used, and study arm and distance to Lake Victoria were included in all models.

#### Target Endpoints

We dichotomized year 5 prevalence at 10%, 25%, and 50% and mean intensity at 5, 10, 25, and 50 EPG. The area under the receiver operating characteristic curves (AUROC) were optimized according to the AUROC [34] to find year 1–4 prevalence or intensity thresholds, mean of years 1 and 2, mean of years 1, 2, and 3, change at year 2 from year 1, and change at 3 from year 1 [35] compared with each dichotomization. Additional analyses using year 1 hotspots and year 5 thresholds from mixture models [36] are included in the Supplementary Materials but will not be discussed here.

### Ethics Statement

The protocol for this study was approved by the National Scientific and Ethical Review Committees of the Kenya Medical Research Institute (SSC no. 1820) and the Institutional Review Boards of the University of Georgia and the Centers for Disease Control and Prevention (protocol number 6016). Adults and parents or guardians of children participating in the study provided written informed consent. Study participants less than 18 years of age provided informed assent. The trial is number 16755535 registered at ISRCTN.

## RESULTS

For all outcomes except mean intensity in 5- to 8-year olds, there were differences in the distributions across years, indicating significant decreases in schistosomiasis burden during the trial (Table 1). At baseline, prevalence and mean intensity were much higher in 9- to 12-year-olds and adults compared with 5- to 8-year-olds. Although, by year 5, each age group had similar medians and ranges.

**Table 1.** Summary Statistics of Village-Level Prevalence (EPG >0), Mean-Intensity, Median-Intensity, and High-Intensity Prevalence (EPG ≥400) by Year

Age Group	Year 1, Median (Range)	Year 2, Median (Range)	Year 3, Median (Range)	Year 4, Median (Range)	Year 5, Median (Range)	*P*
Prevalence						
5- to 8-year-olds	20.34 (0.00–100.00)		12.50 (0.00–88.57)		8.89 (0.00–81.25)	<.001
9- to 12-year-olds	59.47 (8.33–100.00)	42.17 (9.46–98.55)	33.67 (5.41–96.00)	22.00 (4.00–98.00)	19.00 (0.00–99.00)	<.001
Adults	44.68 (6.25–91.84)		12.77 (2.04–55.32)		12.00 (0.00–100.00)	.008
Intensity (mean)						
5- to 8-year-olds	19.72 (0.00–476.47)		6.15 (0.00–351.86)		6.68 (0.00–516.00)	.097
9- to 12-year-olds	50.46 (3.68–454.86)	29.36 (1.73–488.40)	24.31 (1.19–435.76)	11.88 (0.44–201.18)	10.31 (0.00–452.38)	<.001
Adults	50.07 (1.50–270.86)		9.12 (0.26–156.00)		7.92 (0.00–480.96)	<.001
Intensity (median)						
5- to 8-year-olds	0.00 (0.00–432.00)		0.00 (0.00–216.00)		0.00 (0.00–144.00)	<.001
9- to 12-year-olds	8.00 (0.00–426.00)	0.00 (0.00–496.00)	0.00 (0.00–387.00)	0.00 (0.00–96.00)	0.00 (0.00–250.00)	<.001
Adults	0.00 (0.00–198.00)		0.00 (0.00–12.00)		0.00 (0.00–126.00)	<.001
High-Intensity Prevalence						
5- to 8-year-olds	0.00 (0.00–52.94)		0.00 (0.00–36.36)		0.00 (0.00–38.46)	<.001
9- to 12-year-olds	2.86 (0.00–51.00)	2.04 (0.00–60.00)	1.00 (0.00–46.94)	0.00 (0.00–16.00)	0.00 (0.00–38.00)	<.001
Adults	2.13 (0.00–32.65)		0.00 (0.00–17.02)		0.00 (0.00–26.00)	<.001

The Kruskal-Wallis test was used to compare differences across years by age group.

Abbreviations: EPG, eggs per gram.

### Hotspots

**Figure 1.**
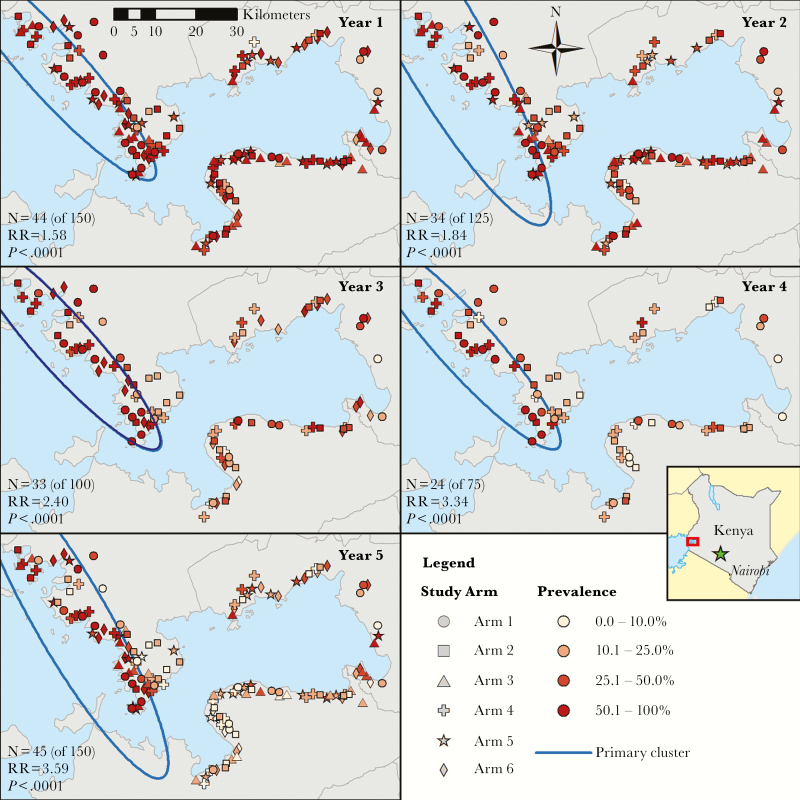
Map of study villages with shapes denoting the study arm and shading by village-level prevalence (percentage of participants with eggs in stool) for 9- to 12-year-old participants and cluster borders. The primary cluster from SaTScan analyses is outlined in blue. Abbreviation: RR, relative risk.

Spatial analyses detected a hotspot in western Siaya County among 9- to 12-year-olds in all 5 years of the trial (Figure 1). The boundaries of the hotspot change slightly over the 5 years but contained approximately one-third of villages in all years. The relative risk comparing the prevalence in villages inside the hotspot to prevalence in villages outside the hotspot increased steadily from 1.58 in year 1 to 3.59 in year 5. There was no association between study arm and hotspot in year 1 for 9- to 12-year-olds (χ^2^_(5)_ = 1.42, *P* = .92) with similar results for each year and age group (Supplementary Table S1).

**Figure 2.**
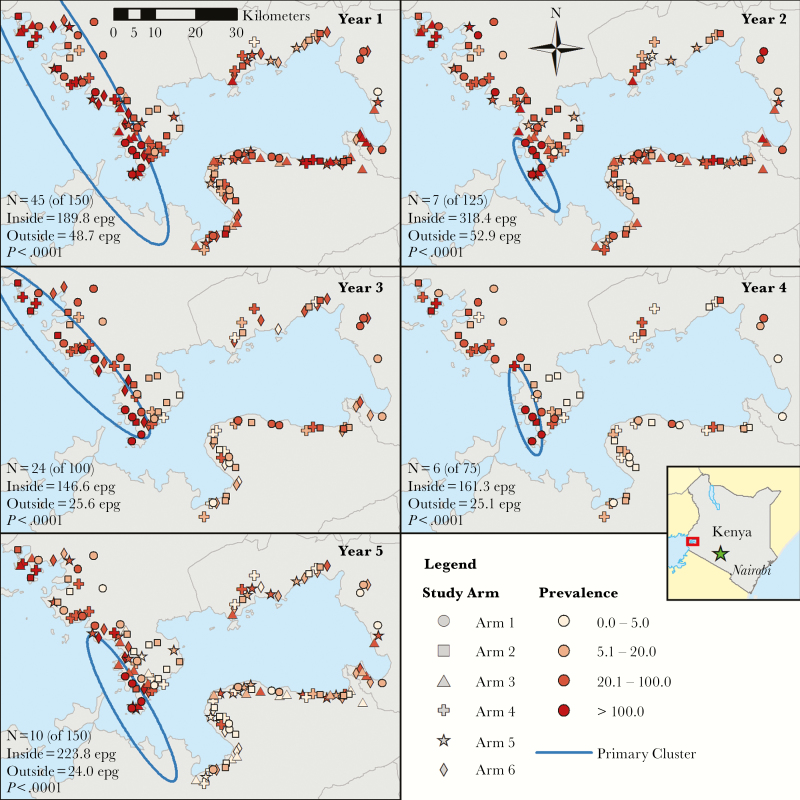
Map of study villages with shapes denoting the study arm and shading by village-level intensity (mean eggs per gram [epg] of stool) for 9- to 12-old participants and cluster borders. The primary cluster from SaTScan analyses is outlined in blue.

Using mean infection intensity among 9- to 12-year-olds resulted in more variability in the shape and size of the hotspot (Figure 2). The number of hotspot villages fell from 45 in year 1 to 10 in year 5. The mean infection intensity inside the hotspot remained similar (year 1 = 189.8 EPG, year 5 = 223.8 EPG) but dropped outside the hotspot from 48.7 EPG in year 1 to 24.0 EPG in year 5.

The presence of the same hotspot was corroborated in multiple analyses (Supplementary Figures S2–S7, S9–S10). Compared to 9- to 12-year-olds, hotspot size and shape were similar when based on prevalence and mean intensity in 5- to 8-year-olds (Supplementary Figures S2 and Figure S5); in many analyses, the size of the hotspot became smaller or disappeared entirely as the study progressed (Supplementary Figures S3–S5, S7, S9). In 2 outcomes (adult mean intensity and adult high-intensity prevalence), there was no hotspot in that location (Supplementary Figures S8, S11).

### Changes From Baseline

There were differences in changes from baseline between hotspot and nonhotspot villages (Table 2). A statistically significant prevalence ratio (PR) was detected by year 2 (PR = 1.15; 95% confidence interval [CI] = 1.05–1.26, *P* = .003), and a statistically significant arithmetic mean ratio (AMR) of mean infection intensity was detected by year 3 (AMR = 1.49; CI = 1.12–1.99, *P* = .007). Ratios greater than 1 indicate that hotspot villages did not decrease as much as nonhotspot villages. Matched analyses showed similar inferences, suggesting the results are not confounded by study arm, baseline prevalence, or distance from Lake Victoria. Hotspot villages also did not decrease as much as nonhotspot villages in 5- to 8-year-olds and adult prevalence, but changes between the hotspot and nonhotspot villages were only associated in the matched data for 5- to 8-year-olds at year 5 (Supplementary Table S2).

**Table 2.** Comparison of Change From Baseline Prevalence and Mean Intensity for Villages Inside the Hotspot at Year 1 to Villages Outside the Hotspot, 9- to 12-Year-Olds

Comparison	All Villages (N = 150)	*P*	Matched Villages (N = 42)	*P*
Prevalence	PR (95% CI)		PR (95% CI)	
Change from year 1 to year 2	1.15 (1.05–1.26)	.0033	1.08 (0.93–1.25)	.2944
Change from year 1 to year 3	1.46 (1.29–1.64)	<.0001	1.49 (1.16–1.93)	.0022
Change from year 1 to year 4	1.90 (1.61–2.24)	<.0001	2.14 (1.65–2.78)	<.0001
Change from year 1 to year 5	2.20 (1.88–2.59)	<.0001	2.28 (1.57–3.31)	<.0001
Mean intensity	AMR (95% CI)		AMR (95% CI)	
Change from year 1 to year 2	1.07 (0.85–1.36)	.5585	0.94 (0.68–1.29)	.7043
Change from year 1 to year 3	1.49 (1.12–1.99)	.0068	2.70 (1.69–4.31)	<.0001
Change from year 1 to year 4	1.68 (1.17–2.42)	.0053	3.13 (1.97–5.00)	<.0001
Change from year 1 to year 5	2.34 (1.70–3.22)	<.0001	2.46 (1.28–4.73)	.007

All 150 villages are included in first set of analyses, whereas matched villages includes villages inside the year 1 hotspot who were paired with a village outside the hotspot with a similar distance to Lake Victoria (<1 km of difference), baseline prevalence (<10% difference), and the same study arm. Results for prevalence analyses are reported as PRs of the change in prevalence from year 1 and as AMRs for the change in mean intensity from year 1.

Abbreviations: AMRs, arithmetic means ratio; CI, confidence interval; PR, prevelance ratio.

### Moderators

Few potential moderators were found (Supplementary Table S3). When using mean intensity as the outcome, and only in the 5- to 8-year-old population, drinking from a river and categorized NDVI (taken either at the school location or the closest shore location to the school) were identified as potential moderators. The NDVI measures the density of vegetation in an area by dividing the proportion of near-infrared light absorbed over the sum of the near-infrared and visible light proportions absorbed. The results suggest that, as vegetation density increases, the ratio of the change from baseline of mean infection intensity decreases when comparing hotspot villages to nonhotspot villages.

Comparing intensity among villages inside the hotspot to those outside, the villages which reported sometimes or never drinking from a river had a higher ratio (AMR = 47.16; 95% CI = 18.03–123.33) than those which reported drinking from the river all the time (AMR = 3.96; 95% CI = 2.50–6.29) or often (AMR = 3.99; 95% CI = 2.34–6.79). For both NDVI variables, AMRs were much higher in villages in the first 2 quartiles of NDVI than those in the higher 2 quartiles (Supplementary Table S3).

### Target Endpoints

The ROC analyses were able to predict whether a village fell below a threshold in year 5 with good accuracy (Table 3 and Supplementary Table S4). Villages falling below 25% prevalence at year 5 could be predicted at year 1 with a sensitivity of 86.36% (95% CI = 78.41–93.18) and a specificity of 74.19% (95% CI = 62.90–83.91). The sensitivity (92%; 95% CI = 86.00–97.00) and specificity (70%; 95% CI = 56.00–82.00) were similar for villages falling below a mean EPG of 25 at year 5. For both measures, using means of multiple years resulted in a similar AUROC to years 1–3, but AUROC was worse when using charge from year 1. Results for other thresholds are included in the Supplementary Materials (Supplementary Table S4).

**Table 3.** Results From ROC Curve Analyses of Whether Villages Fall Below Thresholds of 25% Prevalence and 25 Mean EPG in Year 5 to Predict Prior Years, Means of Prior Years, or Changes in Prior Years

Prevalence				
Year	Area Under the ROC Curve (%)	Threshold (%)	Sensitivity (95% CI)	Specificity (95% CI)
1	84.56	67.38	86.36 (78.41–93.18)	74.19 (62.90–83.91)
2	84.45	53.83	85.53 (77.63–92.14)	79.59 (67.35–89.80)
3	95.10	43.71	89.06 (81.25–95.31)	91.67 (80.56–100.00)
4	97.37	41.73	96.15 (90.38–100.00)	86.96 (73.91–100.00)
5	NA	25.00	NA	NA
Mean of 1 and 2	87.22	58.69	85.84 (81.93–89.46)	83.94 (78.76–89.12)
Mean of 1, 2, and 3	92.73	54.08	88.46 (84.23–91.92)	91.30 (86.09–95.65)
Change at 2 (from 1)	59.76	−16.83	44.88 (39.46–50.00)	76.68 (70.47–82.38)
Change at 3 (from 1)	78.40	−16.27	69.93 (64.86–75.34)	79.87 (73.38–86.36)
Intensity				
Year	Area Under the ROC Curve (%)	Threshold (EPG)	Sensitivity (95% CI)	Specificity (95% CI)
1	86.38	113.43	92.00 (86.00–97.00)	70.00 (56.00–82.00)
2	82.46	67.91	88.10 (80.95–94.05)	70.73 (56.10–85.37)
3	90.03	64.67	91.67 (84.72–97.22)	75.00 (57.14–89.29)
4	86.65	37.26	91.07 (83.93–98.21)	84.21 (68.42–100.00)
5	NA	25.00	NA	NA
Mean of 1 and 2	84.40	71.00	84.62 (80.77–88.19)	73.29 (66.46–79.50)
Mean of 1, 2, and 3	86.00	104.11	94.64 (92.13–97.14)	68.42 (58.95–76.84)
Change at 2 (from 1)	59.54	−37.20	79.67 (75.27–83.79)	49.07 (40.99–56.52)
Change at 3 (from 1)	59.28	−72.39	89.02 (85.37–92.38)	47.54 (38.52–56.56)

The area under the ROC curve can be thought of as the expected percentage that a randomly drawn village below the year 5 threshold is less than a randomly drawn village that is above the threshold.

Abbreviations: CI, confidence interval; EPG, eggs per gram; NA, nonapplicable; ROC, receiving operating characteristic.

## DISCUSSION

We identified a persistent, burden hotspot [37] in villages on the western edge of the study area throughout the trial. Only 1 other study has evaluated the presence of persistent hotspots of schistosomiasis [38], and few studies have explored whether infectious disease hotspots change over time [39]. Persistent hotspots are recognized as having a disproportionate influence on driving transmission in infectious diseases [40], which means their discovery can be useful for disease control. We determined hotspots by statistical significance and allowed the extent of the hotspot to be determined by robust methods [29, 30], which has been recommended [37]. Predefined thresholds of changes over time are easier to use, but establishing a unilateral threshold to define hotspots in multiple locations may be challenging due to differences in baseline infection levels, environmental factors, local behaviors, and other factors. Hence, methods that make a relative comparison may be more useful across different locations and diseases, even though such definitions may not be robust [38]. Hopefully, the use of these methods in other contexts will result in an operational definition for persistent hotspots.

Overall prevalence and intensity of schistosomiasis decreased during the trial, although this was moderated in some circumstances by whether the villages was part of the hotspot. Analyses of the data from 5- to 8-year-olds and 9- to 12-year-olds suggest that prevalence in hotspot villages did not decrease as much as prevalence in nonhotspot villages. Differences between hotspot and nonhotspot village were more pronounced in infection prevalence because all other outcomes suggested a contraction in size. The greater reduction in intensity can be understood based on the association between cure rate and infection intensity, that is, the effects of mass chemoprophylaxis would be felt first on intensity and second on prevalence [41, 42]. Hotspot villages may take longer to reach specific reductions in infections and infection intensity, or, to reach specific reductions, more frequent chemoprophylaxis or additional interventions need to be implemented in hotspot areas. In contrast, decreases in infection prevalence and intensity in adults were not moderated by the hotspot. This may be related to adults having greater resistance to reinfection or less contact with infectious water than the younger age groups and suggests that the effect of annual chemoprophylaxis in adults is similar in villages inside a hotspot compared with those outside.

Our analyses did not identify factors that might moderate the relationship between the hotspot and changes in prevalence and intensity. Analyses suggested that vegetation levels and tendencies of drinking from a river rather than the lake may moderate the overall relationship between mean intensity and the hotspot. Both are plausible moderators because some *S. mansoni* infections have been found along rivers or streams just south of this hotspot [43], and vegetation indices are commonly used for the prediction of human schistosomiasis because they may serve as a proxy for the suitability of snail habitats [44]. Further research will be needed to find possible causes for the persistence of a hotspot. Some possible explanations for the hotspot’s existence and persistence include the presence of superspreaders, differences in snail populations (such as numbers of snails or differences in snail species), and differences in the effectiveness of praziquantel. Superspreaders are individuals that can contribute 80% of infections while consisting of only 20% of the population [45]. The potential impact of superspreaders on preventative chemotherapy interventions for schistosomiasis is a concern [1]. Targeting interventions to superspreaders can result in dramatic improvements in disease control [46]. Evidence of repeated infections occurring in a subpopulation of this area exists [47] and may be continuing to occur. Alternatively, the snail populations might differ between the water contact areas inside and outside the hotspot. The shoreline adjacent to the persistently high prevalence hotspot is along the main body of Lake Victoria, whereas the villages that were more responsive to MDA are distributed around the Winam Gulf. The differences in the ecology of these 2 areas could influence the numbers or relative distribution of appropriate intermediate host snail species, which could result in differences in the force of transmission between the 2 areas. Finally, if the schistosomes infecting people within the hotspot had decreased susceptibility to praziquantel, they may persist where the parasites of the villages outside the hotspot were more readily killed by MDA. Ongoing studies are designed to explore these possibilities.

Finally, we tried to predict which villages would meet specific endpoints by year 5 of the trial. Our findings suggest that villages’ endpoints can be predicted with moderate to high accuracy based on their prevalence and intensity from prior years. Analyses of combined years found that multiple years of data may not be necessary to predict year 5 endpoints, which can save resources. If these results can be applied to other settings, then the thresholds found in these analyses can be used to determine whether villages will fall below, for instance, 25% schistosomiasis prevalence after 4 years of annual chemoprophylaxis. The prediction improved over time; hence, after implementation of a schistosomiasis control program, continued monitoring of program data is needed to amend the strategy for areas with reduced MDA effectiveness. This concurs with mathematical models that have shown spatially-targeted schistosomiasis interventions with a geostatistical approach to be more cost effective than other approaches [48]. Adapting control programs based on the setting are recommended [1, 49] and may decrease program costs [50]. In addition, the scan statistic produces a dichotomy of hotspot or nonhotspot; that delineation may be suboptimal as gradations in risk and relative changes year to year [38] may be more important for control programs.

We were unable to identify explanations for the presence and persistence of the hotspot. Minimal covariate information at the individual level was collected during this trial. We used the distance to the closest shore point as a proxy for water contact, but this alone may not represent a village’s risk. Furthermore, the shore locations may not represent the actual distance people travel to the lake or where individuals experience their water contact. The information at the village level was all self-reported and retrospective and those data may suffer from multiple biases. Finally, the remote sensing data were minimally variable across this study area. Data at higher resolutions may provide more detailed and accurate results. Further data, especially data on risk behaviors at the individual level, will need to be collected on this area to determine why this hotspot persists.

## CONCLUSIONS

Despite these shortcomings, these analyses provide a detailed comparison between a persistent hotspot of *S. mansoni* infection and a nonhotspot area during the first 5 years of chemoprophylaxis. These analyses show that the effects of a MDA are heterogeneous across this study area and a persistent hotspot may not experience the same benefit as nonhotspot areas. If reliable, these thresholds could then be used to determine villages that may not reach program endpoints before 5 years, allowing for the possibility to change intervention strategy to reach a target endpoint.

## Supplementary Data

Supplementary materials are available at *The Journal of Infectious Diseases* online. Consisting of data provided by the authors to benefit the reader, the posted materials are not copyedited and are the sole responsibility of the authors, so questions or comments should be addressed to the corresponding author.

## Supplementary Material

Supplementary Material

## Notes

***Acknowledgments.*** This work is published with permission of the Director of the Kenya Medical Research Institute.

***Financial support.*** This work was funded by the University of Georgia Research Foundation, Inc., which was funded by the Bill & Melinda Gates Foundation for the SCORE project.

***Potential conflicts of interest.*** All authors: No reported conflicts of interest. All authors have submitted the ICMJE Form for Disclosure of Potential Conflicts of Interest. Conflicts that the editors consider relevant to the content of the manuscript have been disclosed.
