# Errata

**DOI:** 10.1093/infdis/jiy146

**Published:** 2018-04-09

“A Persistent Hotspot of Schistosoma mansoni Infection in a Five-Year Randomized Trial of Praziquantel Preventative Chemotherapy Strategies” by Wiegand et al. [J Infect Dis 2017; 216(12): 1425–33]. This is an Open Access article and should have contained the following copyright line: © The Author [2018]. Published by Oxford University Press for the Infectious Diseases Society of America. This is an Open Access article distributed under the terms of the Creative Commons Attribution License (http://creativecommons.org/licenses/by/4.0/), which permits unrestricted reuse, distribution, and reproduction in any medium, provided the original work is properly cited.

The authors regret this error.

