# Supplementary material for: A Persistent Hotspot of Schistosoma mansoni Infection in a Five-Year Randomized Trial of Praziquantel Preventative Chemotherapy Strategies
Source: J Infect Dis. 2017 Sep 16;216(11):1425–33. doi: 10.1093/infdis/jix496 (PMC5913648; doi:10.1093/infdis/jix496)

Supplementary Material

**Article title**: A persistent hotspot of *Schistosoma mansoni* infection in a five-year randomized trial of

praziquantel preventative chemotherapy strategies

Contents

[Supplementary Tables 4](#_Toc492037142)

[**Table S1**. Associations between study arm and whether or not villages were inside the high prevalence or intensity focus. 4](#_Toc492037143)

[**Table S2**. Comparison of change from baseline prevalence and mean intensity for villages inside the baseline focus to villages outside the focus, 5-8 year olds and Adults. Results for prevalence analyses are reported as prevalence ratios (PRs) of the change in prevalence from year 1 and as arithmetic mean ratios (AMRs) for the change in mean intensity from year 1. 5](#_Toc492037144)

[**Table S3**. Variables with a significant interaction with year 1 focus variable. Results are from negative binomial regression models with generalized estimating equations to account for multiple observations per village. Results are reported as arithmetic mean ratios (AMRs) of communities inside the focus compared to those outside the focus by level of the covariate. 6](#_Toc492037145)

[**Table S4**. Results from receiver operating characteristic (ROC) curve analyses of whether villages fall below a variety of thresholds for prevalence mean intensity in year 5 based on prior years, means of prior years, or changes in prior years. The area under the ROC curve can be thought of as the expected percentage that a randomly drawn village below the year 5 threshold is less than a randomly drawn village that above the threshold. 7](#_Toc492037146)

[Supplementary Figures 10](#_Toc492037147)

[**Figure S1**. SCORE study trial diagram. 10](#_Toc492037148)

[**Figure S2**. Map of study villages with shapes denoting the study arm and shading by village-level prevalence (percentage of participants with eggs in stool) for 5-8 year old participants and cluster borders. The blue lines are the primary clusters from SaTScan analyses. 11](#_Toc492037149)

[**Figure S3**. Map of study villages with shapes denoting the study arm and shading by village-level prevalence (percentage of participants with eggs in stool) for adult participants and cluster borders. The blue lines are the primary clusters from SaTScan analyses. 12](#_Toc492037150)

[**Figure S4**. Map of study villages with shapes denoting the study arm and shading by village-level intensity (median eggs per gram of stool) for 9-12 year old participants and cluster borders. The blue lines are the primary clusters from SaTScan analyses. Relevant secondary clusters are in green. 13](#_Toc492037151)

[**Figure S5**. Map of study villages with shapes denoting the study arm and shading by village-level intensity (mean eggs per gram of stool) for 5-8 year old participants and cluster borders. The blue lines are the primary clusters from SaTScan analyses. 14](#_Toc492037152)

[**Figure S6**. Map of study villages with shapes denoting the study arm and shading by village-level intensity (mean eggs per gram of stool) for adult participants and cluster borders. The blue lines are the primary clusters from SaTScan analyses. Relevant secondary clusters are in green. 15](#_Toc492037153)

[**Figure S7**. Map of study villages with shapes denoting the study arm and shading by village-level intensity (median eggs per gram of stool) for 5-8 year old participants and cluster borders. The blue lines are the primary clusters from SaTScan analyses. Relevant secondary clusters are in green. 16](#_Toc492037154)

[**Figure S8**. Map of study villages with shapes denoting the study arm and shading by village-level intensity (median eggs per gram of stool) for adult participants and cluster borders. The blue lines are the primary clusters from SaTScan analyses. Relevant secondary clusters are in green. 17](#_Toc492037155)

[**Figure S9**. Map of study villages with shapes denoting the study arm and shading by village-level prevalence of high-intensity infections (percentage of participants with ≥ 400 eggs per gram of stool) for 9-12 year old participants and cluster borders. The blue lines are the primary clusters from SaTScan analyses. 18](#_Toc492037156)

[**Figure S10**. Map of study villages with shapes denoting the study arm and shading by village-level prevalence of high-intensity infections (percentage of participants with ≥ 400 eggs per gram of stool) for 5-8 year old participants and cluster borders. The blue lines are the primary clusters from SaTScan analyses. 19](#_Toc492037157)

[**Figure S11**. Map of study villages with shapes denoting the study arm and shading by village-level prevalence of high-intensity infections (percentage of participants with ≥ 400 eggs per gram of stool) for adult participants and cluster borders. The blue lines are the primary clusters from SaTScan analyses. Relevant secondary clusters are in green. 20](#_Toc492037158)

[**Figure S12**. Village-level prevalence (left) and mean intensity (right) distributions with estimated density functions from Gaussian mixture models and cutoff point from where the density functions intersect. 21](#_Toc492037159)

[**Figure S13**. ROC curves for determination of optimal cutoff at year 1 for prevalence (top panel) and mean intensity (bottom panel) using multiple thresholds. 22](#_Toc492037160)

[**Figure S14**. ROC curves for determination of optimal cutoff at year 2 for prevalence (top panel) and mean intensity (bottom panel) using multiple thresholds. 23](#_Toc492037161)

[**Figure S15**. ROC curves for determination of optimal cutoff at year 3 for prevalence (top panel) and mean intensity (bottom panel) using multiple thresholds. 24](#_Toc492037162)

[**Figure S16**. ROC curves for determination of optimal cutoff at year 4 for prevalence (top panel) and mean intensity (bottom panel) using multiple thresholds. 25](#_Toc492037163)

[**Figure S17**. ROC curves for determination of optimal cutoff at year 5 for prevalence (top panel) and mean intensity (bottom panel) using multiple thresholds. 26](#_Toc492037164)

[**Figure S18**. ROC curves for determination of optimal cutoff using the mean of years 1 and 2 for prevalence (top panel) and mean intensity (bottom panel) using multiple thresholds. 27](#_Toc492037165)

[**Figure S19**. ROC curves for determination of optimal cutoff using the mean of years 1, 2, and 3 for prevalence (top panel) and mean intensity (bottom panel) using multiple thresholds. 28](#_Toc492037166)

[**Figure S20**. ROC curves for determination of optimal cutoff using the change at year 2 from year 1 for prevalence (top panel) and mean intensity (bottom panel) using multiple thresholds. 29](#_Toc492037167)

[**Figure S21**. ROC curves for determination of optimal cutoff using the change at year 3 from year 1 for prevalence (top panel) and mean intensity (bottom panel) using multiple thresholds. 30](#_Toc492037168)

# Supplementary Tables

### **Table S1**. Associations between study arm and whether or not villages were inside the high prevalence or intensity focus.

| Measure | Age Group | Year | Arm 1  N (%) | Arm 2  N (%) | Arm 3  N (%) | Arm 4  N (%) | Arm 5  N (%) | Arm 6  N (%) | p |
| --- | --- | --- | --- | --- | --- | --- | --- | --- | --- |
| Prevalence | 5-8 year olds | 1 | 8 (32) | 6 (24) | 5 (20) | 7 (28) | 5 (20) | 6 (24) | 0.92 |
|  | 5-8 year olds | 3 | 11 (46) | 6 (25) |  | 8 (32) |  | 9 (36) | 0.49 |
|  | 5-8 year olds | 5 | 10 (40) | 6 (24) | 7 (28) | 7 (28) | 7 (28) | 8 (32) | 0.87 |
|  | 9-12 year olds | 1 | 9 (36) | 6 (24) | 8 (32) | 8 (32) | 6 (24) | 7 (28) | 0.92 |
|  | 9-12 year olds | 2 | 9 (36) | 6 (24) | 6 (24) | 7 (28) | 6 (24) |  | 0.85 |
|  | 9-12 year olds | 3 | 10 (40) | 7 (28) |  | 8 (32) |  | 8 (32) | 0.84 |
|  | 9-12 year olds | 4 | 10 (40) | 6 (24) |  | 8 (32) |  |  | 0.48 |
|  | 9-12 year olds | 5 | 10 (40) | 6 (24) | 7 (28) | 7 (28) | 7 (28) | 8 (32) | 0.87 |
|  | Adults | 1 | 10 (40) | 6 (24) | 7 (28) | 7 (28) | 7 (28) | 7 (28) | 0.88 |
|  | Adults | 3 | 9 (38) | 6 (24) |  |  |  |  | 0.31 |
|  | Adults | 5 | 3 (13) | 1 (4) | 1 (4) | 0 (0) | 2 (8) | 0 (0) | 0.20* |
| Mean | 5-8 year olds | 1 | 5 (20) | 1 (4) | 3 (12) | 0 (0) | 3 (12) | 2 (8) | 0.20* |
| Intensity | 5-8 year olds | 3 | 8 (33) | 6 (25) |  | 7 (28) |  | 6 (24) | 0.89 |
|  | 5-8 year olds | 5 | 4 (16) | 1 (4) | 2 (8) | 0 (0) | 2 (8) | 1 (4) | 0.38* |
|  | 9-12 year olds | 1 | 10 (40) | 6 (24) | 7 (28) | 7 (28) | 7 (28) | 8 (32) | 0.87 |
|  | 9-12 year olds | 2 | 3 (12) | 1 (4) | 1 (4) | 0 (0) | 2 (8) |  | 0.60* |
|  | 9-12 year olds | 3 | 8 (32) | 5 (20) |  | 5 (20) |  | 6 (24) | 0.73 |
|  | 9-12 year olds | 4 | 4 (16) | 1 (4) |  | 1 (4) |  |  | 0.35* |
|  | 9-12 year olds | 5 | 4 (16) | 1 (4) | 2 (8) | 0 (0) | 2 (8) | 1 (4) | 0.38* |
|  | Adults | 1 | 0 (0) | 3 (12) | 5 (20) | 3 (12) | 3 (12) | 6 (24) | 0.14* |
|  | Adults | 3 | 1 (4) | 0 (0) |  |  |  |  | 0.49* |
|  | Adults | 5 | 0 (0) | 0 (0) | 0 (0) | 0 (0) | 1 (4) | 0 (0) | 1.00* |
| Median | 5-8 year olds | 1 | 5 (20) | 1 (4) | 3 (12) | 0 (0) | 3 (12) | 1 (4) | 0.13* |
| Intensity | 5-8 year olds | 3 | 1 (4) | 0 (0) |  | 0 (0) |  | 0 (0) | 0.49* |
|  | 5-8 year olds | 5 | 0 (0) | 0 (0) | 0 (0) | 0 (0) | 0 (0) | 1 (4) | 1.00* |
|  | 9-12 year olds | 1 | 8 (32) | 6 (24) | 5 (20) | 7 (28) | 5 (20) | 6 (24) | 0.92 |
|  | 9-12 year olds | 2 | 3 (12) | 1 (4) | 1 (4) | 0 (0) | 2 (8) |  | 0.60* |
|  | 9-12 year olds | 3 | 0 (0) | 1 (4) |  | 0 (0) |  | 0 (0) | 1.00* |
|  | 9-12 year olds | 4 | 5 (20) | 1 (4) |  | 0 (0) |  |  | 0.04* |
|  | 9-12 year olds | 5 | 4 (16) | 1 (4) | 2 (8) | 0 (0) | 2 (8) | 1 (4) | 0.38* |
|  | Adults | 1 | 0 (0) | 0 (0) | 0 (0) | 1 (4) | 0 (0) | 0 (0) | 1.00* |
|  | Adults | 3 | 0 (0) | 1 (4) |  |  |  |  | 1.00* |
|  | Adults | 5 | 0 (0) | 0 (0) | 0 (0) | 0 (0) | 1 (4) | 0 (0) | 1.00* |

Notes: * Test used Fisher’s Exact Test. All other tests use Pearson’s Chi-squared test.

Focus definitions are at the year being tested; hence, the focus definition for the mean intensity at year 4 for 9-12 year olds is the focus at that year.

### **Table S2**. Comparison of change from baseline prevalence and mean intensity for villages inside the baseline focus to villages outside the focus, 5-8 year olds and Adults. Results for prevalence analyses are reported as prevalence ratios (PRs) of the change in prevalence from year 1 and as arithmetic mean ratios (AMRs) for the change in mean intensity from year 1.

| **5-8 year olds** | Full data |  | Matched data |  |
| --- | --- | --- | --- | --- |
|  | (N=150) |  | (N=40) |  |
| Prevalence comparison | PR (95% CI) | p | PR (95% CI) | p |
| Change from year 1 to year 3 | 1.35 (1.02, 1.78) | 0.037 | 1.06 (0.92, 1.22) | 0.4423 |
| Change from year 1 to year 5 | 1.51 (1.19, 1.92) | 0.0007 | 1.53 (1.20, 1.97) | 0.0007 |
|  |  |  |  |  |
| Mean intensity comparison | AMR (95% CI) | p | AMR (95% CI) | p |
| Change from year 1 to year 3 | 1.32 (0.75, 2.31) | 0.3312 | 0.88 (0.68, 1.15) | 0.355 |
| Change from year 1 to year 5 | 1.02 (0.61, 1.71) | 0.9301 | 2.65 (1.67, 4.20) | <.0001 |
|  |  |  |  |  |
| **Adults** | Full data |  | Matched data |  |
|  | (N=150) |  | (N=62) |  |
| Prevalence comparison | PR (95% CI) | p | PR (95% CI) | p |
| Change from year 1 to year 3 | 1.31 (0.93, 1.85) | 0.1259 | 1.30 (0.83, 2.02) | 0.2553 |
| Change from year 1 to year 5 | 1.36 (1.03, 1.80) | 0.0288 | 1.37 (0.90, 2.09) | 0.1385 |
|  |  |  |  |  |
| Mean intensity comparison | AMR (95% CI) | p | AMR (95% CI) | p |
| Change from year 1 to year 3 | 1.49 (0.75, 2.96) | 0.2498 | 0.68 (0.27, 1.76) | 0.4315 |
| Change from year 1 to year 5 | 1.77 (0.99, 3.17) | 0.0552 | 1.36 (0.71, 2.58) | 0.3505 |

### **Table S3**. Variables with a significant interaction with year 1 focus variable. Results are from negative binomial regression models with generalized estimating equations to account for multiple observations per village. Results are reported as arithmetic mean ratios (AMRs) of communities inside the focus compared to those outside the focus by level of the covariate.

| 5-8 year olds |  |  |  |
| --- | --- | --- | --- |
| *Intensity* |  |  |  |
| Variable | Level | AMR (95% CI) | p |
| Drinking from a river | All the time | 3.96 (2.50, 6.29) | <.0001 |
|  | Often | 3.99 (2.34, 6.79) | <.0001 |
|  | Sometimes/never | 47.16 (18.03, 123.33) | <.0001 |
| NDVI (closest shore location) | Q1: 0-0.40 | 7.91 (4.83, 12.95) | <.0001 |
|  | Q2: 0.40-0.53 | 6.54 (3.45, 12.38) | <.0001 |
|  | Q3: 0.53-0.59 | 1.87 (1.14, 3.04) | 0.0125 |
|  | Q4: 0.59-0.79 | 1.77 (0.89, 3.53) | 0.1063 |
| NDVI (school location) | Q1: 0.20-0.52 | 4.59 (2.62, 8.02) | <.0001 |
|  | Q2: 0.52-0.55 | 9.18 (4.03, 20.88) | <.0001 |
|  | Q3: 0.55-0.58 | Non-estimable |  |
|  | Q4: 0.58-0.67 | 2.04 (1.11, 3.77) | 0.0223 |

Notes: p-values test whether PR or AMR is different from 1. Continuous variables are split into quartiles and Q1, Q2, Q3, and Q4 denote the first, second, third, and fourth quartiles, respectively. An attempt was made to correct NDVI values that clearly fell into Lake Victoria, but some may still be over water.

### **Table S4**. Results from receiver operating characteristic (ROC) curve analyses of whether villages fall below a variety of thresholds for prevalence mean intensity in year 5 based on prior years, means of prior years, or changes in prior years. The area under the ROC curve can be thought of as the expected percentage that a randomly drawn village below the year 5 threshold is less than a randomly drawn village that above the threshold.

| **Prevalence** |  |  |  |  |
| --- | --- | --- | --- | --- |
| Year | Area under the ROC curve (%) | Threshold (%) | Sensitivity (95% CI) | Specificity (95% CI) |
| Year 5 prevalence < 50% | | | | |
| 1 | 93.21 | 76.53 | 87.61 (81.42-93.81) | 91.89 (81.08-100.00) |
| 2 | 91.25 | 52.54 | 73.96 (64.58-82.29) | 96.55 (89.66-100.00) |
| 3 | 97.43 | 58.46 | 88.31 (80.52-94.81) | 95.65 (86.96-100.00) |
| 4 | 97.5 | 41.73 | 88.33 (80.00-96.67) | 100.00 (100.00-100.00) |
| 5 | NA | 50 | NA | NA |
| Mean of 1 and 2 | 94.15 | 61.22 | 79.90 (75.98-83.82) | 97.44 (94.02-100.00) |
| Mean of 1, 2, and 3 | 97.33 | 74.53 | 96.67 (94.67-98.67) | 86.67 (78.67-93.33) |
|  | 97.33 | 60.13 | 83.33 (79.00-87.33) | 100.00 (100.00-100.00) |
| Change at 2 (from 1) | 63.18 | -5.20 | 72.30 (67.65-76.47) | 53.85 (45.28-62.39) |
| Change at 3 (from 1) | 78.94 | -15.07 | 67.24 (62.39-72.08) | 84.85 (77.78-90.93) |
| Year 5 prevalence < 10% | | | | |
| 1 | 74.28 | 67.38 | 90.91 (81.82-100.00) | 47.01 (37.61-55.56) |
| 2 | 79.78 | 41.01 | 89.66 (79.31-100.00) | 63.54 (54.14-72.92) |
| 3 | 85.64 | 35.22 | 96.30 (88.89-100.00) | 63.01 (52.05-73.97) |
| 4 | 89.59 | 26.00 | 100.00 (100.00-100.00) | 65.38 (51.92-78.85) |
| 5 | NA | 10 | NA | NA |
| Mean of 1 and 2 | 78.60 | 56.92 | 96.24 (92.48-99.25) | 54.08 (49.23-58.93) |
| Mean of 1, 2, and 3 | 81.35 | 41.40 | 86.96 (80.00-93.04) | 65.38 (59.62-71.15) |
| Change at 2 (from 1) | 60.92 | -29.72 | 24.81 (17.29-32.33) | 95.92 (93.88-97.71) |
| Change at 3 (from 1) | 68.84 | -34.44 | 44.09 (35.43-52.78) | 86.07 (82.35-89.78) |
| Inclusion in Year 1 prevalence hotspot | | | | |
| 1 | 88.63 | 70.92 | 84.09 (75.00-93.18) | 83.96 (76.42-90.57) |
| 2 | 85.21 | 54.62 | 83.78 (70.27-94.59) | 79.55 (70.45-87.50) |
| 3 | 90.62 | 58.46 | 83.33 (70.00-96.67) | 91.43 (84.29-97.14) |
| 4 | 92.98 | 33.50 | 91.30 (78.26-100.00) | 90.38 (82.64-98.08) |
| 5 | 92.65 | 32.50 | 86.36 (75.00-95.45) | 89.62 (83.96-95.28) |
| Mean of 1 and 2 | 87.56 | 66.59 | 78.34 (71.34-84.08) | 85.33 (81.52-88.86) |
| Mean of 1, 2, and 3 | 89.13 | 60.13 | 82.61 (75.65-89.57) | 88.46 (84.62-92.31) |
| Change at 2 (from 1) | 58.77 | -16.74 | 81.53 (75.16-87.26) | 45.65 (40.49-50.28) |
| Change at 3 (from 1) | 70.20 | -20.02 | 85.29 (79.41-91.18) | 57.01 (51.59-62.42) |
| Less than mixture model derived cutoff (27.82%) of Year 5 prevalence | | | | |
| 1 | 86.24 | 70.92 | 87.23 (79.79-93.62) | 75.00 (62.50-85.71) |
| 2 | 85.52 | 53.83 | 81.71 (73.17-90.24) | 81.40 (69.77-93.02) |
| 3 | 94.28 | 43.71 | 85.07 (76.12-92.54) | 90.91 (81.74-100.00) |
| 4 | 98.18 | 41.73 | 94.55 (87.27-100.00) | 95.00 (85.00-100.00) |
| 5 | NA | 27.82 | NA | NA |
| Mean of 1 and 2 | 88.17 | 61.22 | 84.55 (80.90-87.93) | 83.43 (77.51-88.76) |
| Mean of 1, 2, and 3 | 92.27 | 60.13 | 87.27 (83.27-90.91) | 90.00 (84.00-96.00) |
| Change at 2 (from 1) | 58.03 | -11.35 | 60.39 (55.06-65.73) | 60.95 (53.85-68.05) |
| Change at 3 (from 1) | 75.85 | -16.27 | 66.56 (61.41-71.70) | 77.70 (70.50-84.17) |
|  |  |  |  |  |
| **Intensity** |  |  |  |  |
| Year | Area under the ROC curve (%) | Threshold (EPG) | Sensitivity (95% CI) | Specificity (95% CI) |
| Year 5 mean infection intensity < 50 EPG | | | | |
| 1 | 95.48 | 117.04 | 86.18 (80.47-91.87) | 88.89 (74.07-100.00) |
| 2 | 92.88 | 87.63 | 90.20 (84.31-95.10) | 86.96 (73.91-100.00) |
| 3 | 97.92 | 71.00 | 89.29 (82.14-95.24) | 100.00 (100.00-100.00) |
| 4 | 98.02 | 61.32 | 92.06 (84.13-98.41) | 100.00 (100.00-100.00) |
| 5 | NA | 50 | NA | NA |
| Mean of 1 and 2 | 95.69 | 128.00 | 95.60 (93.75-97.45) | 82.80 (74.19-90.32) |
| Mean of 1, 2, and 3 | 98.15 | 111.95 | 96.83 (94.60-98.73) | 91.67 (85.00-98.33) |
| Change at 2 (from 1) | 57.42 | -95.95 | 99.31 (98.38-100.00) | 40.86 (31.18-50.54) |
| Change at 3 (from 1) | 66.99 | -72.39 | 86.77 (83.33-90.21) | 61.11 (50.00-72.22) |
| Year 5 mean infection intensity < 10 EPG | | | | |
| 1 | 79.39 | 56.54 | 78.38 (68.92-86.52) | 73.68 (63.16-82.89) |
| 2 | 79.25 | 63.58 | 90.91 (83.33-96.97) | 59.32 (47.46-71.19) |
| 3 | 85.02 | 33.59 | 88.89 (79.63-96.30) | 71.74 (58.70-84.78) |
| 4 | 82.83 | 37.26 | 97.83 (93.48-100.00) | 68.97 (51.72-86.21) |
| 5 | NA | 10 | NA | NA |
| Mean of 1 and 2 | 81.44 | 33.18 | 65.52 (60.00-71.03) | 85.96 (81.28-90.21) |
| Mean of 1, 2, and 3 | 83.58 | 58.38 | 89.13 (84.78-93.04) | 65.52 (57.24-73.12) |
| Change at 2 (from 1) | 58.70 | -34.45 | 79.66 (74.82-84.48) | 44.68 (38.30-50.64) |
| Change at 3 (from 1) | 56.77 | -41.36 | 79.13 (74.40-84.25) | 49.49 (42.35-56.63) |
| Year 5 mean infection intensity < 5 EPG | | | | |
| 1 | 83.28 | 54.65 | 90.00 (82.00-98.00) | 68.00 (58.00-77.00) |
| 2 | 85.11 | 29.23 | 91.11 (82.22-97.78) | 73.75 (63.75-82.53) |
| 3 | 85.37 | 20.48 | 86.11 (75.00-97.22) | 76.56 (65.62-85.94) |
| 4 | 82.81 | 15.51 | 93.55 (83.87-100.00) | 68.18 (54.55-81.82) |
| 5 | NA | 5 | NA | NA |
| Mean of 1 and 2 | 86.25 | 39.39 | 86.80 (82.23-91.37) | 73.78 (68.90-78.35) |
| Mean of 1, 2, and 3 | 88.64 | 46.17 | 96.77 (93.55-99.35) | 70.45 (64.09-76.36) |
| Change at 2 (from 1) | 56.44 | -34.45 | 87.31 (82.74-91.88) | 42.38 (37.20-47.26) |
| Change at 3 (from 1) | 61.62 | -31.81 | 80.59 (74.71-86.47) | 53.93 (48.56-59.64) |
| Inclusion in Year 1 mean infection intensity hotspot | | | | |
| 1 | 88.76 | 113.43 | 75.56 (62.22-86.67) | 91.43 (85.71-96.19) |
| 2 | 85.26 | 56.19 | 81.08 (67.57-91.89) | 84.09 (76.14-90.91) |
| 3 | 92.1 | 42.16 | 87.10 (74.19-96.77) | 91.30 (84.06-97.10) |
| 4 | 91.64 | 37.26 | 82.61 (65.22-95.65) | 96.15 (90.38-100.00) |
| 5 | 92.13 | 27.68 | 80.00 (68.89-91.11) | 90.48 (84.76-95.24) |
| Mean of 1 and 2 | 87.88 | 59.11 | 83.44 (77.69-89.17) | 82.07 (77.99-86.14) |
| Mean of 1, 2, and 3 | 89.05 | 58.38 | 82.61 (75.65-89.57) | 90.38 (86.54-93.85) |
| Change at 2 (from 1) | 59.57 | -57.88 | 35.67 (28.66-43.31) | 96.47 (94.57-98.10) |
| Change at 3 (from 1) | 58.19 | -58.20 | 48.92 (40.29-56.83) | 86.82 (82.96-90.35) |
| Less than mixture model derived cutoff (38.00 EPG) of Year 5 mean infection intensity | | | | |
| 1 | 91.48 | 117.04 | 87.93 (81.90-93.97) | 79.41 (64.71-91.18) |
| 2 | 92.44 | 87.63 | 91.84 (85.71-96.94) | 81.48 (66.67-92.59) |
| 3 | 95.25 | 71.00 | 91.25 (85.00-97.50) | 90.00 (75.00-100.00) |
| 4 | 98.01 | 61.32 | 93.55 (87.10-98.39) | 100.00 (100.00-100.00) |
| 5 | NA | 38.00 | NA | NA |
| Mean of 1 and 2 | 93.84 | 71.00 | 81.34 (77.51-85.17) | 89.72 (84.11-95.33) |
| Mean of 1, 2, and 3 | 97.39 | 58.38 | 82.26 (77.74-86.45) | 100.00 (100.00-100.00) |
| Change at 2 (from 1) | 52.89 | -95.95 | 99.28 (98.33-100.00) | 35.51 (26.17-44.86) |
| Change at 3 (from 1) | 63.72 | -72.39 | 87.91 (84.62-91.21) | 58.14 (47.67-68.60) |
|  |  |  |  |  |

# Supplementary Figures

### **Figure S1**. SCORE study trial diagram.


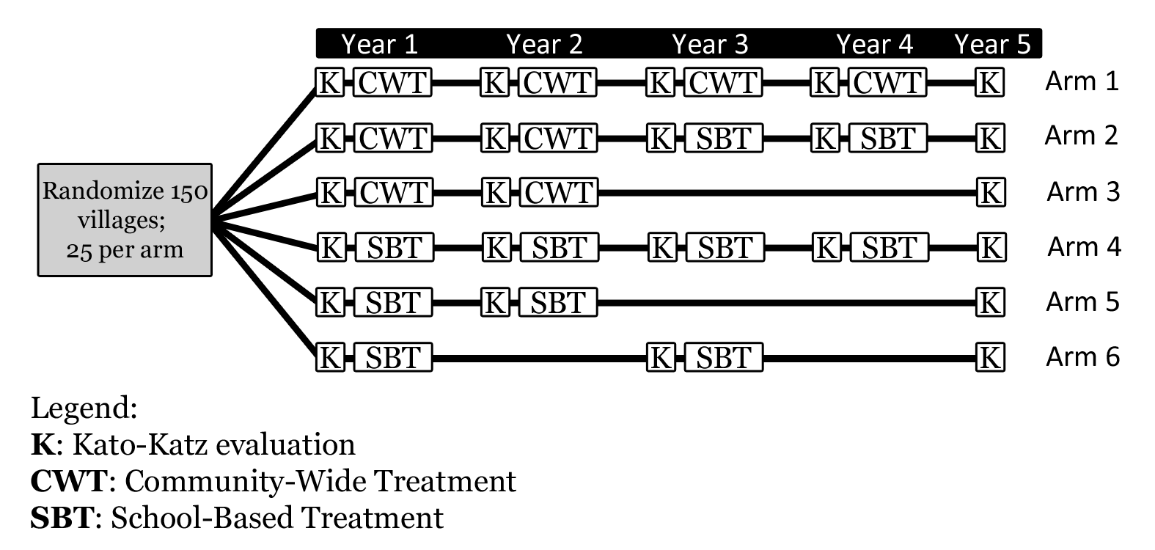


### **Figure S2**. Map of study villages with shapes denoting the study arm and shading by village-level prevalence (percentage of participants with eggs in stool) for 5-8 year old participants and cluster borders. The blue lines are the primary clusters from SaTScan analyses.


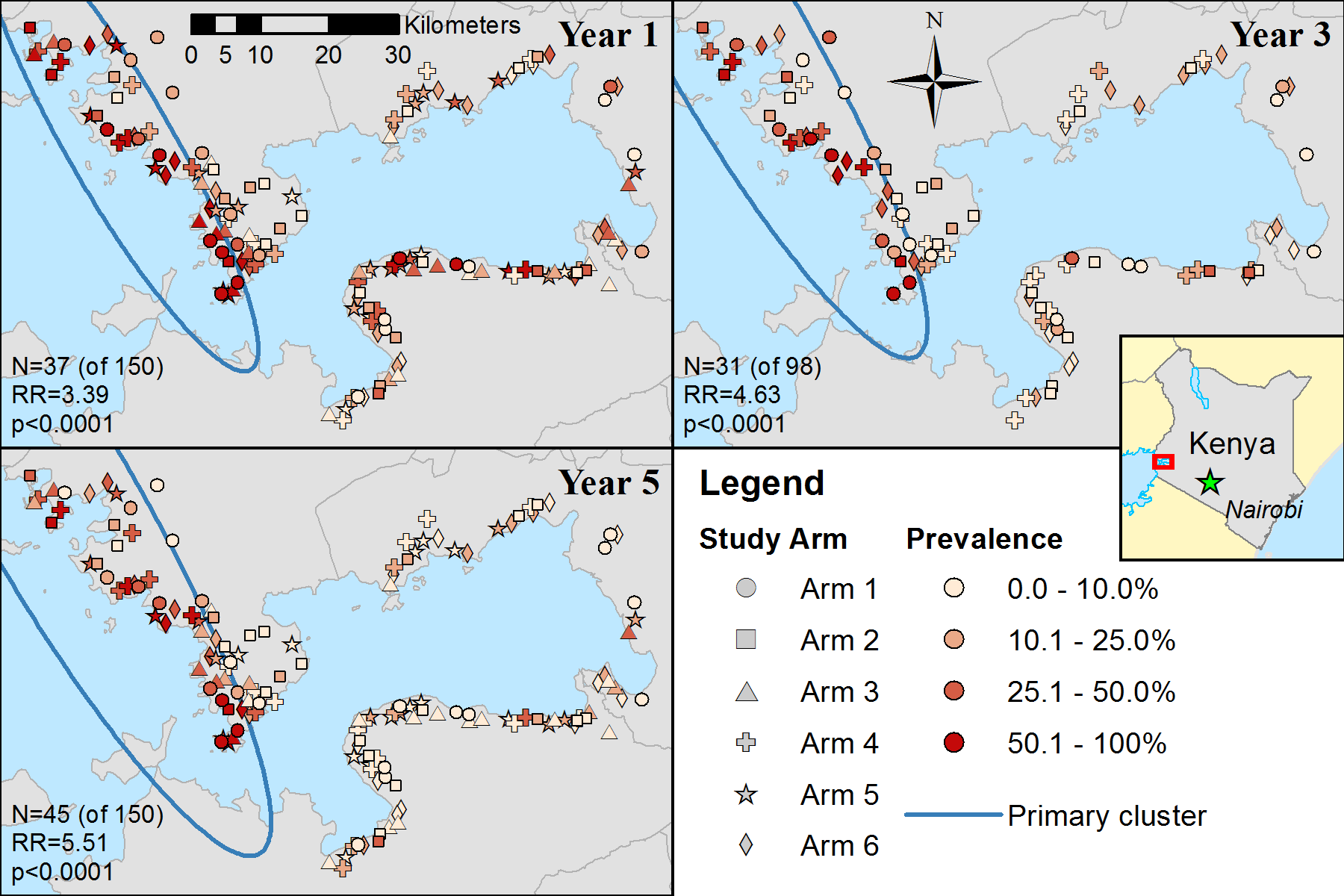


### **Figure S3**. Map of study villages with shapes denoting the study arm and shading by village-level prevalence (percentage of participants with eggs in stool) for adult participants and cluster borders. The blue lines are the primary clusters from SaTScan analyses.


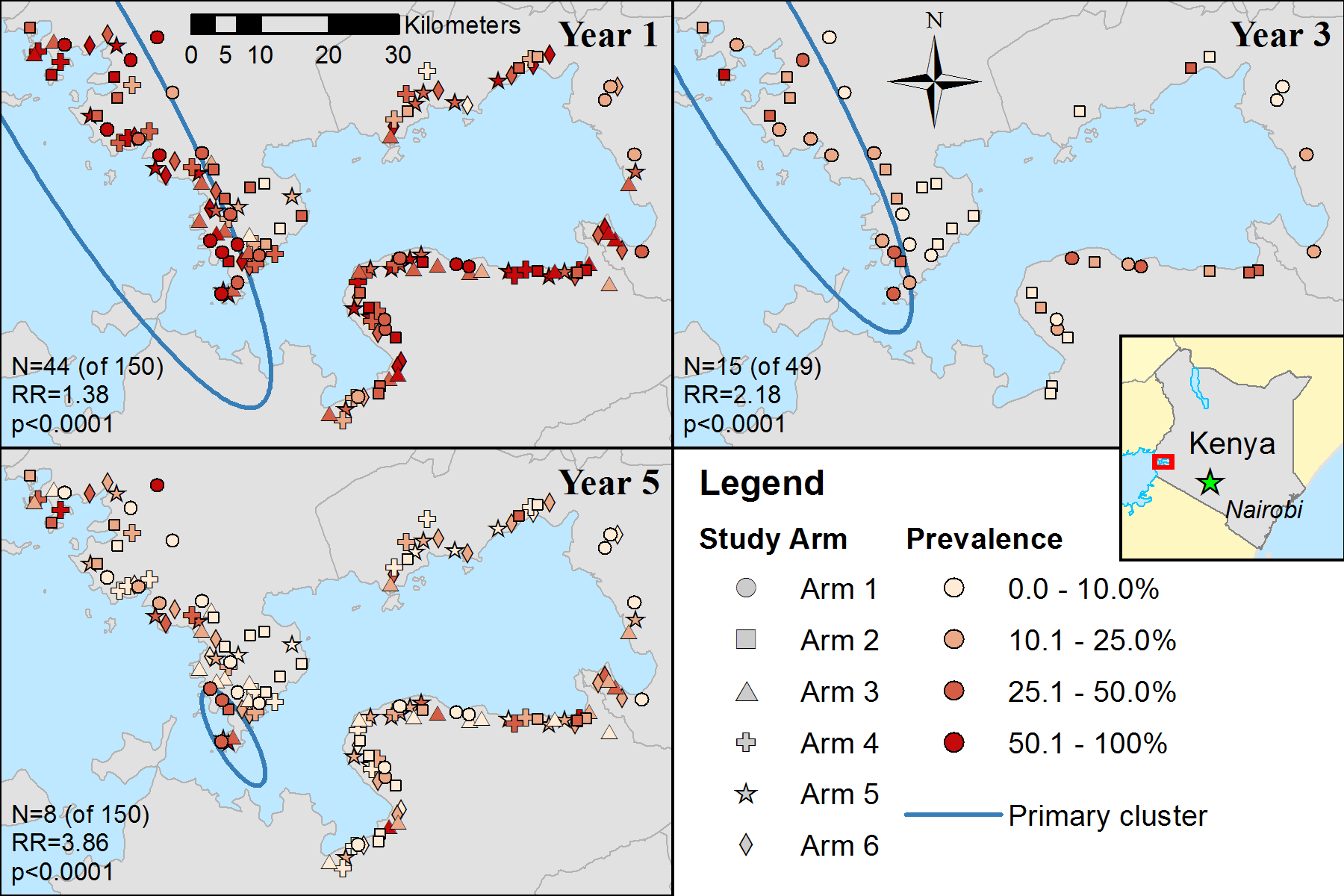


### **Figure S4**. Map of study villages with shapes denoting the study arm and shading by village-level intensity (median eggs per gram of stool) for 9-12 year old participants and cluster borders. The blue lines are the primary clusters from SaTScan analyses. Relevant secondary clusters are in green.


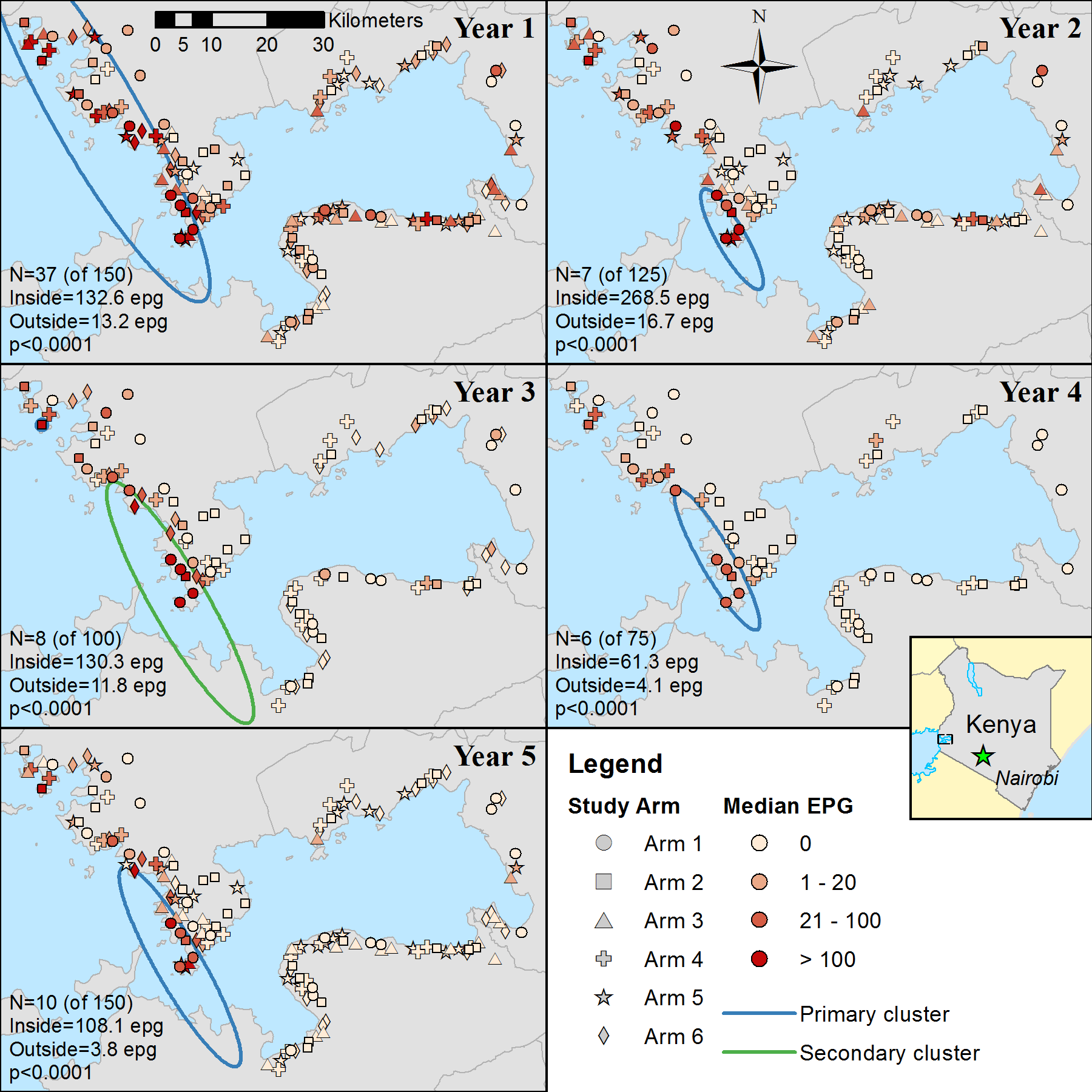


### **Figure S5**. Map of study villages with shapes denoting the study arm and shading by village-level intensity (mean eggs per gram of stool) for 5-8 year old participants and cluster borders. The blue lines are the primary clusters from SaTScan analyses.


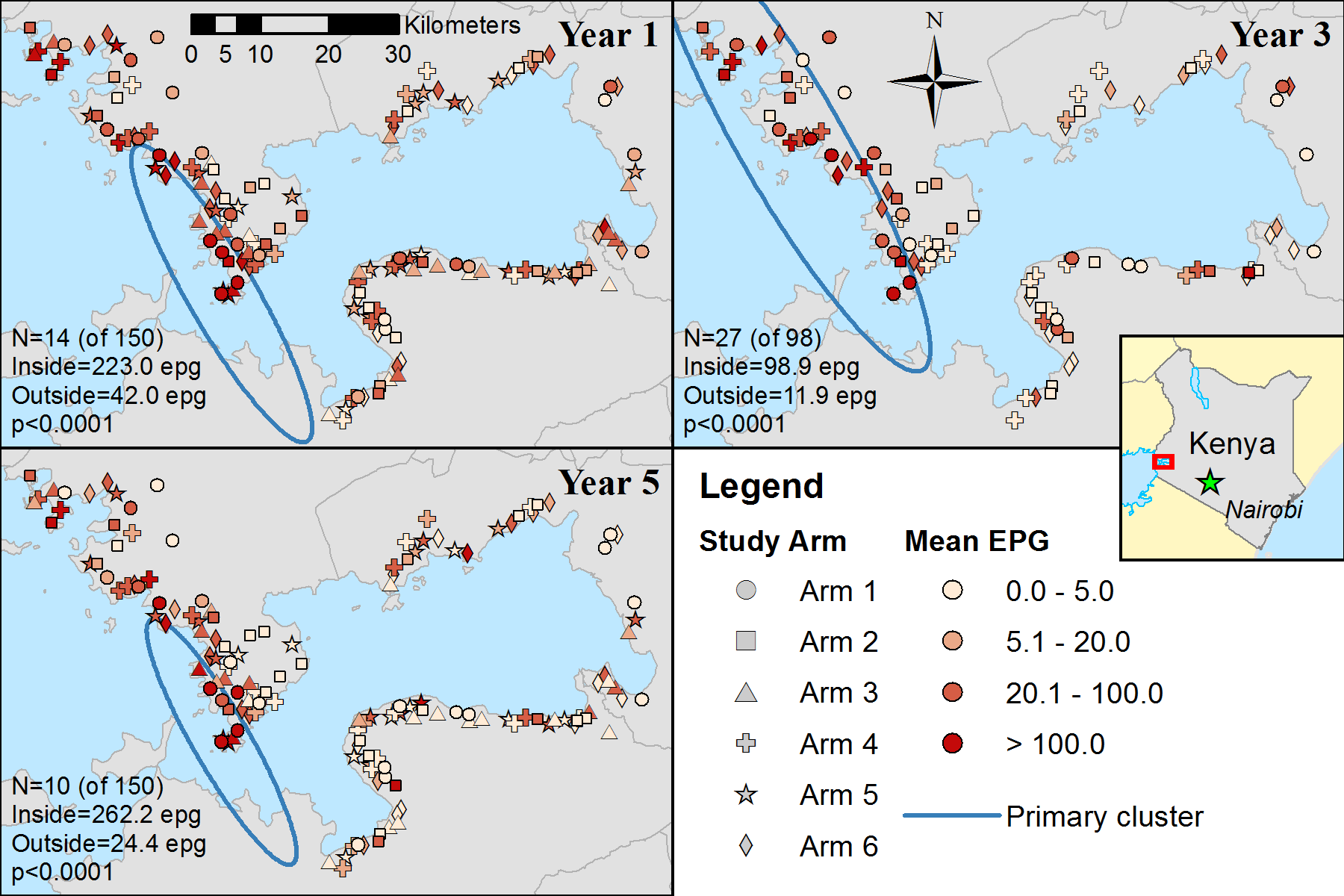


### **Figure S6**. Map of study villages with shapes denoting the study arm and shading by village-level intensity (mean eggs per gram of stool) for adult participants and cluster borders. The blue lines are the primary clusters from SaTScan analyses. Relevant secondary clusters are in green.


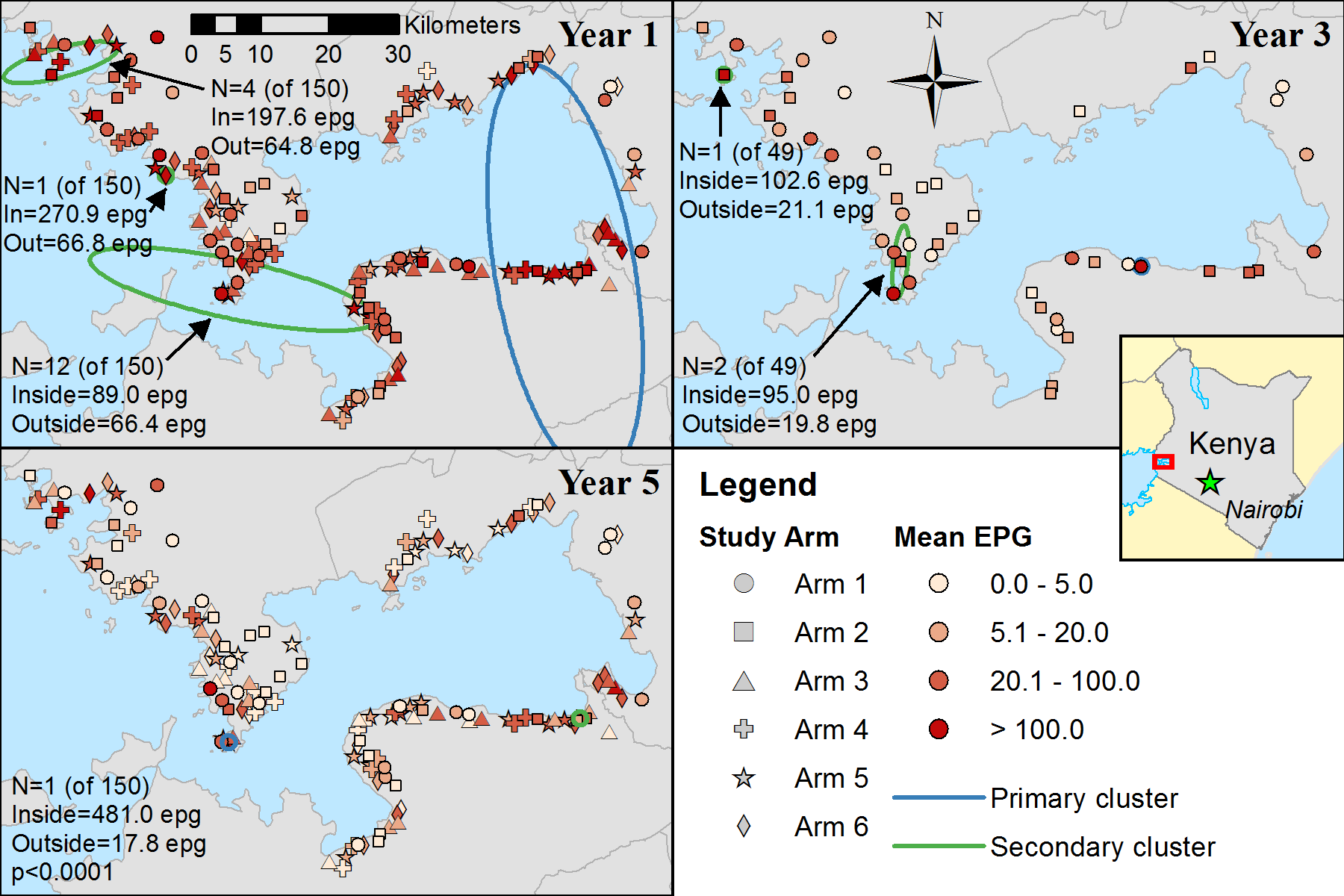


### **Figure S7**. Map of study villages with shapes denoting the study arm and shading by village-level intensity (median eggs per gram of stool) for 5-8 year old participants and cluster borders. The blue lines are the primary clusters from SaTScan analyses. Relevant secondary clusters are in green.


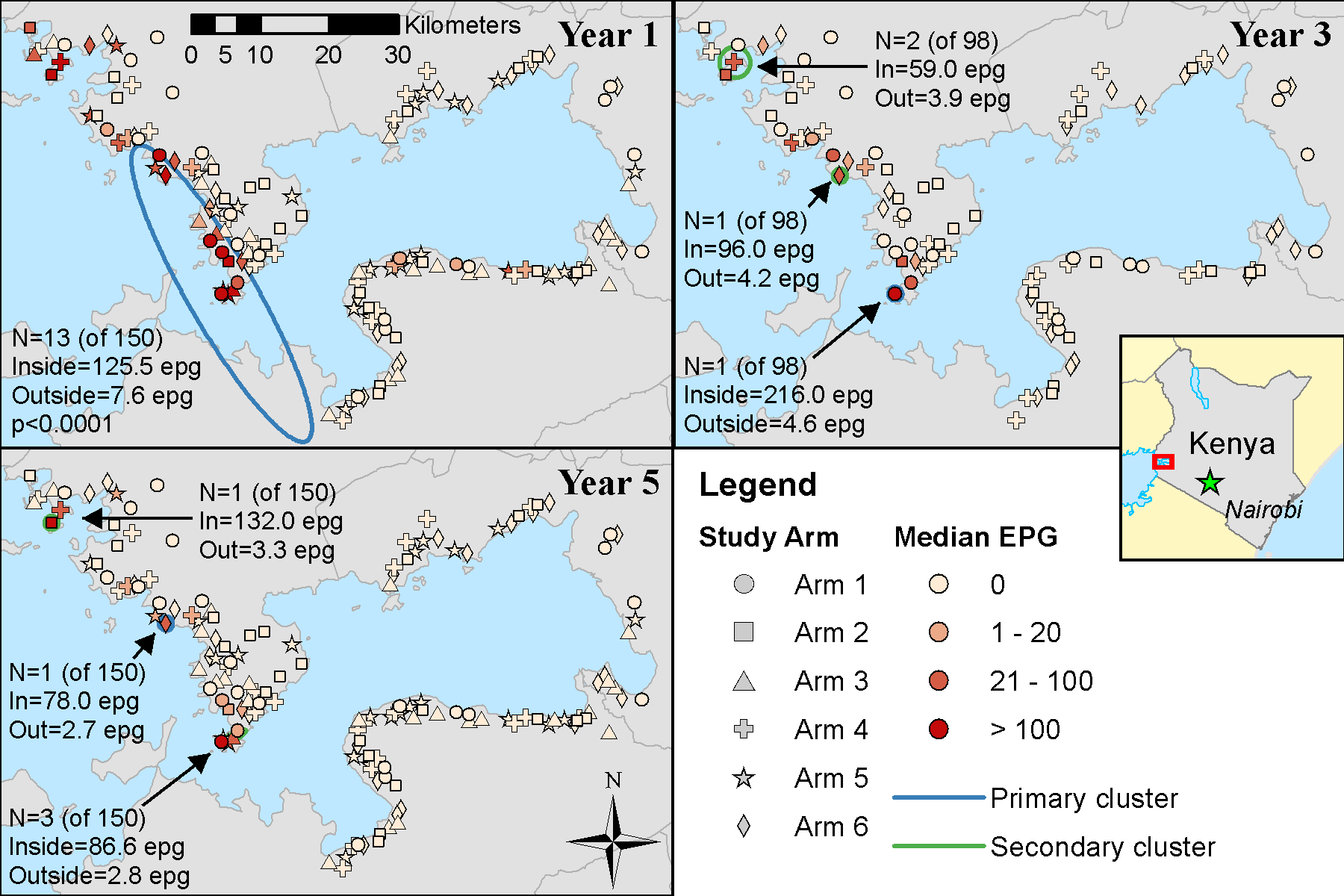


### **Figure S8**. Map of study villages with shapes denoting the study arm and shading by village-level intensity (median eggs per gram of stool) for adult participants and cluster borders. The blue lines are the primary clusters from SaTScan analyses. Relevant secondary clusters are in green.


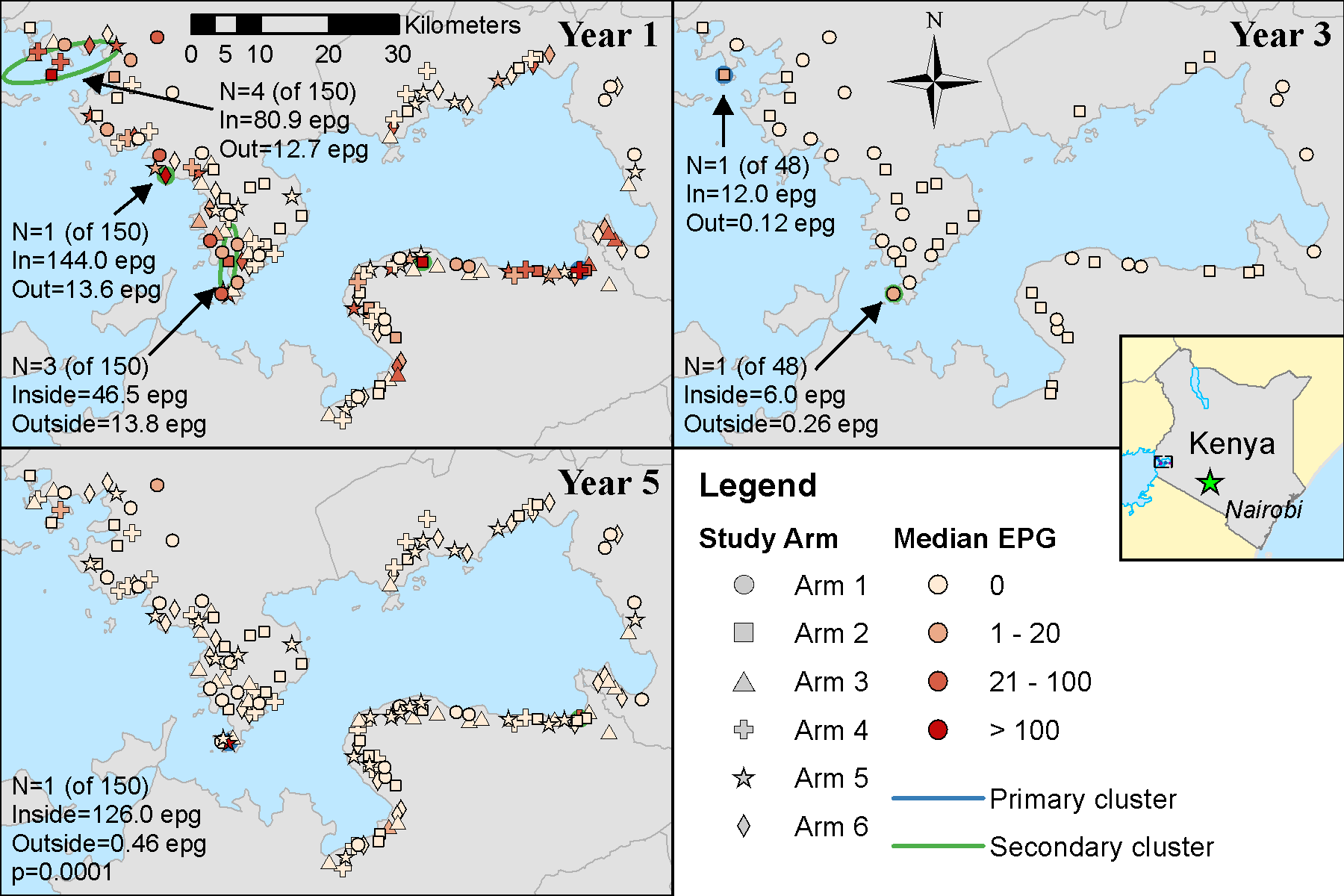


### **Figure S9**. Map of study villages with shapes denoting the study arm and shading by village-level prevalence of high-intensity infections (percentage of participants with ≥ 400 eggs per gram of stool) for 9-12 year old participants and cluster borders. The blue lines are the primary clusters from SaTScan analyses.


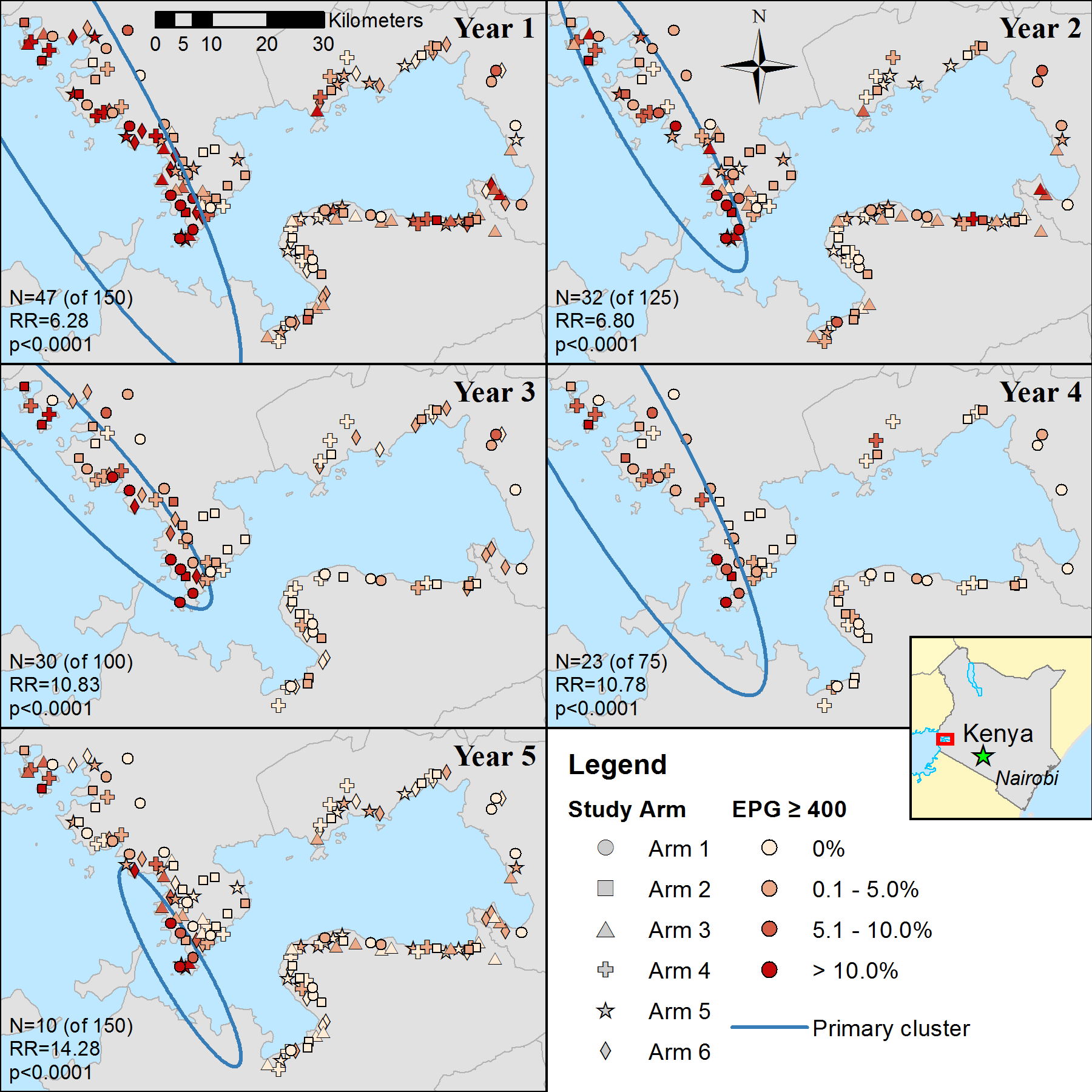


### **Figure S10**. Map of study villages with shapes denoting the study arm and shading by village-level prevalence of high-intensity infections (percentage of participants with ≥ 400 eggs per gram of stool) for 5-8 year old participants and cluster borders. The blue lines are the primary clusters from SaTScan analyses.


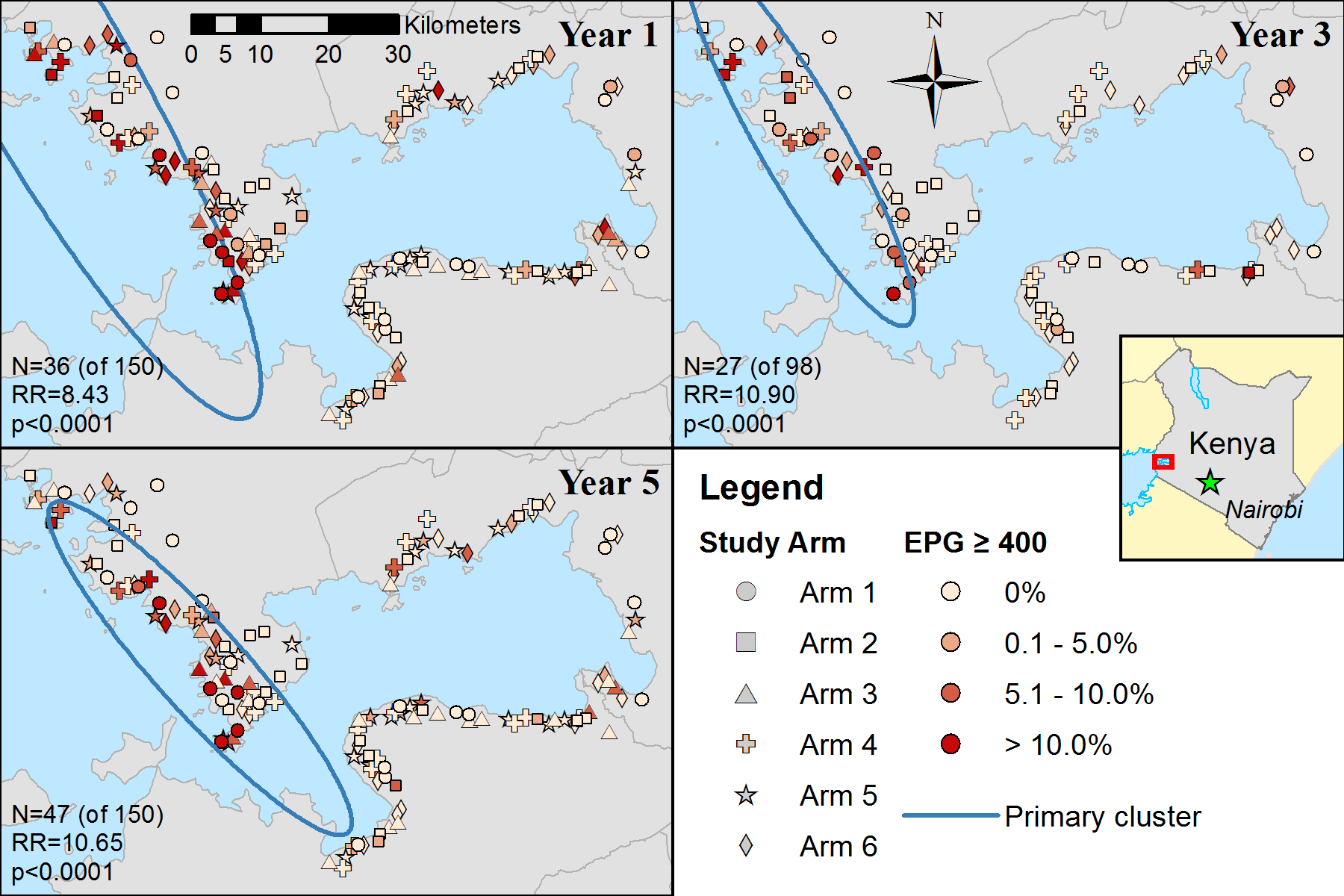


### **Figure S11**. Map of study villages with shapes denoting the study arm and shading by village-level prevalence of high-intensity infections (percentage of participants with ≥ 400 eggs per gram of stool) for adult participants and cluster borders. The blue lines are the primary clusters from SaTScan analyses. Relevant secondary clusters are in green.


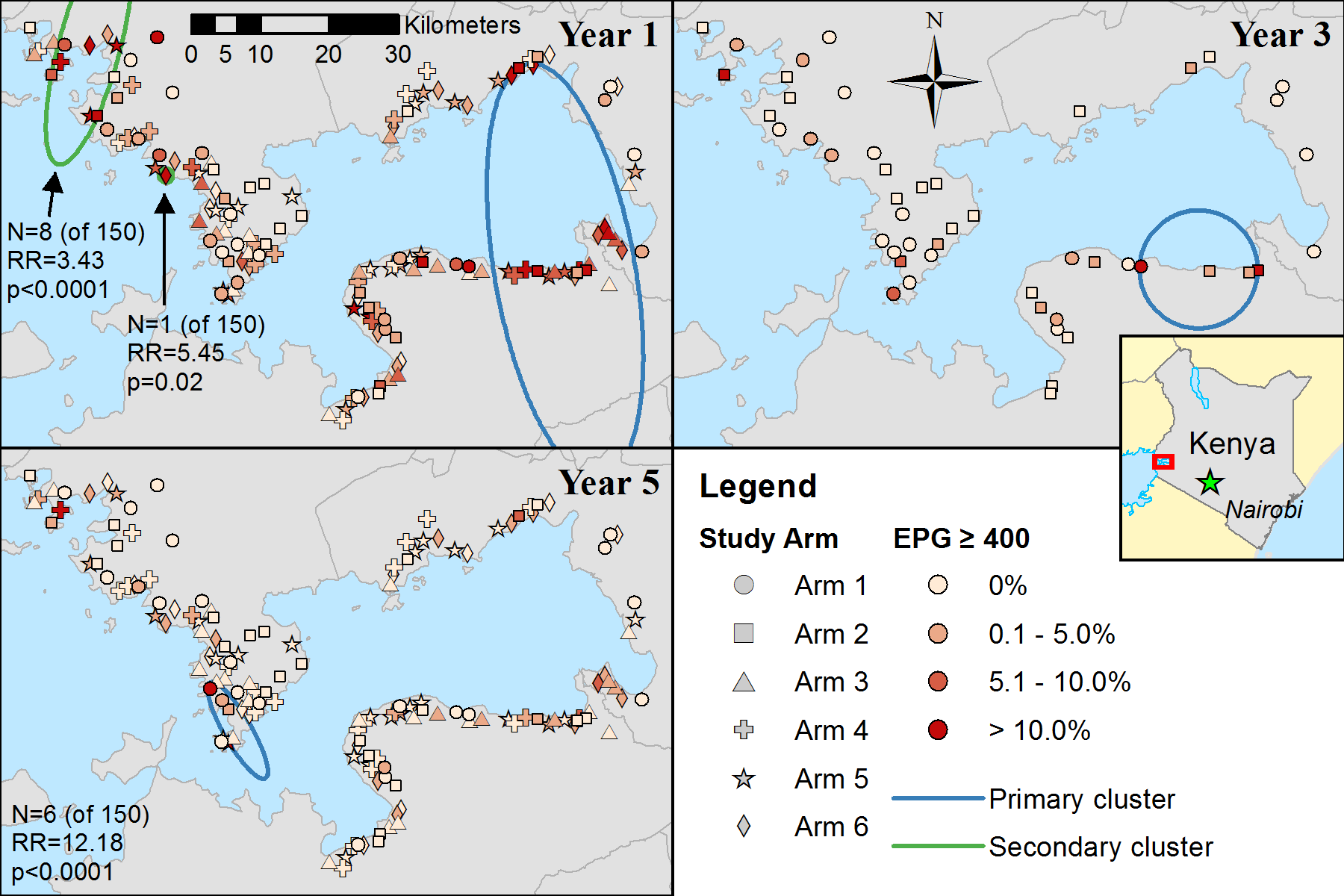


### **Figure S12**. Village-level prevalence (left) and mean intensity (right) distributions with estimated density functions from Gaussian mixture models and cutoff point from where the density functions intersect.


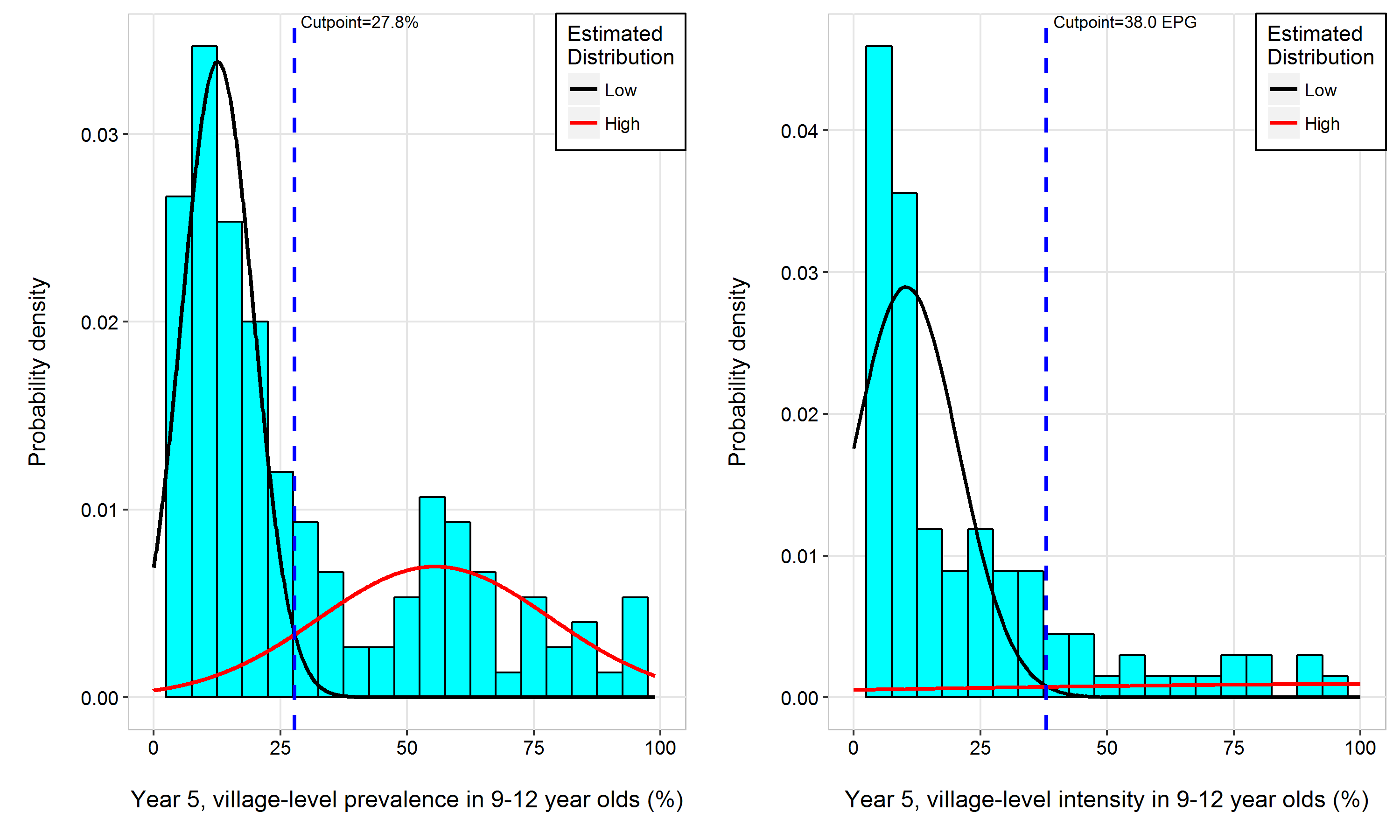


### **Figure S13**. ROC curves for determination of optimal cutoff at year 1 for prevalence (top panel) and mean intensity (bottom panel) using multiple thresholds.


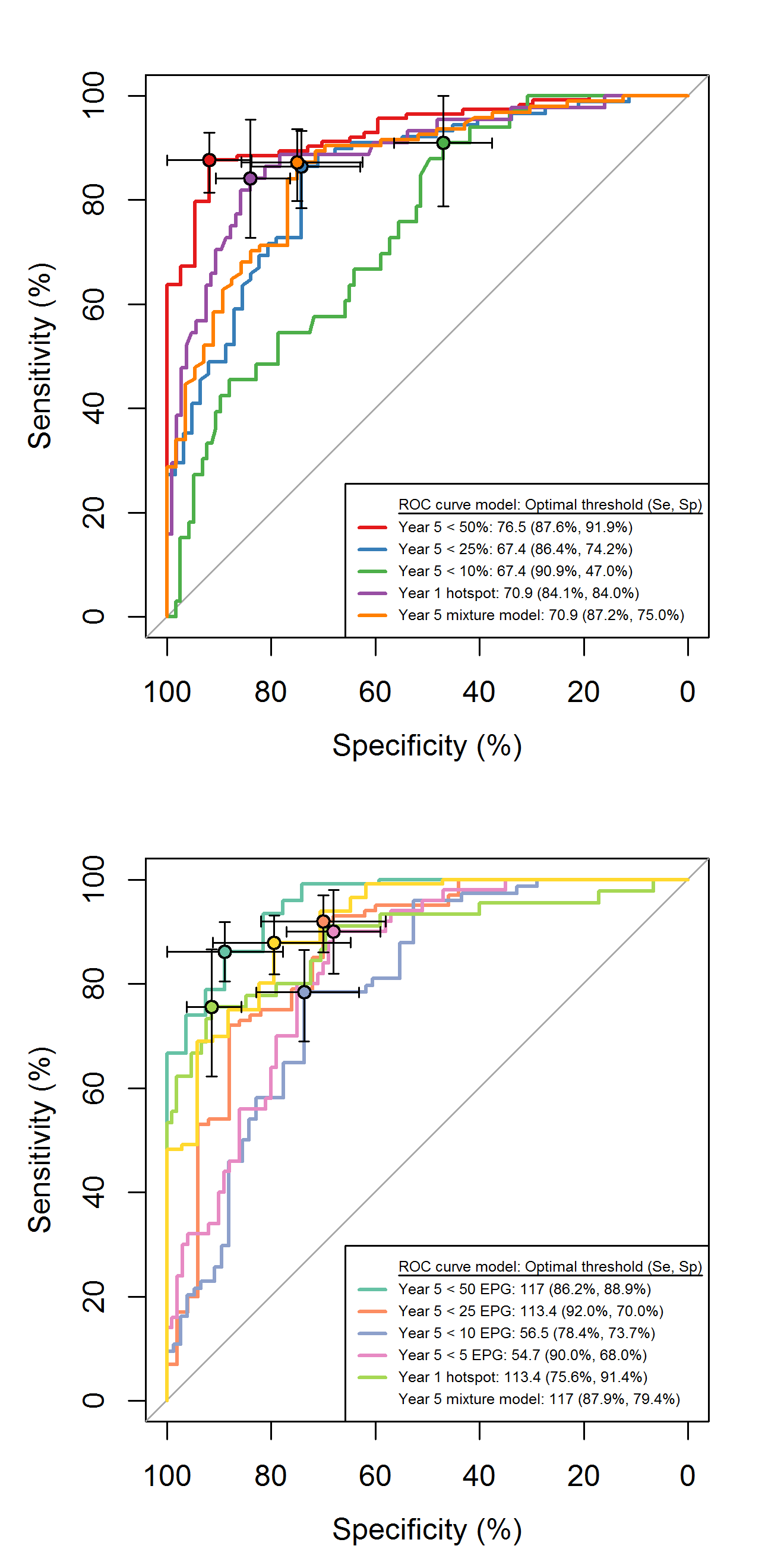


### **Figure S14**. ROC curves for determination of optimal cutoff at year 2 for prevalence (top panel) and mean intensity (bottom panel) using multiple thresholds.


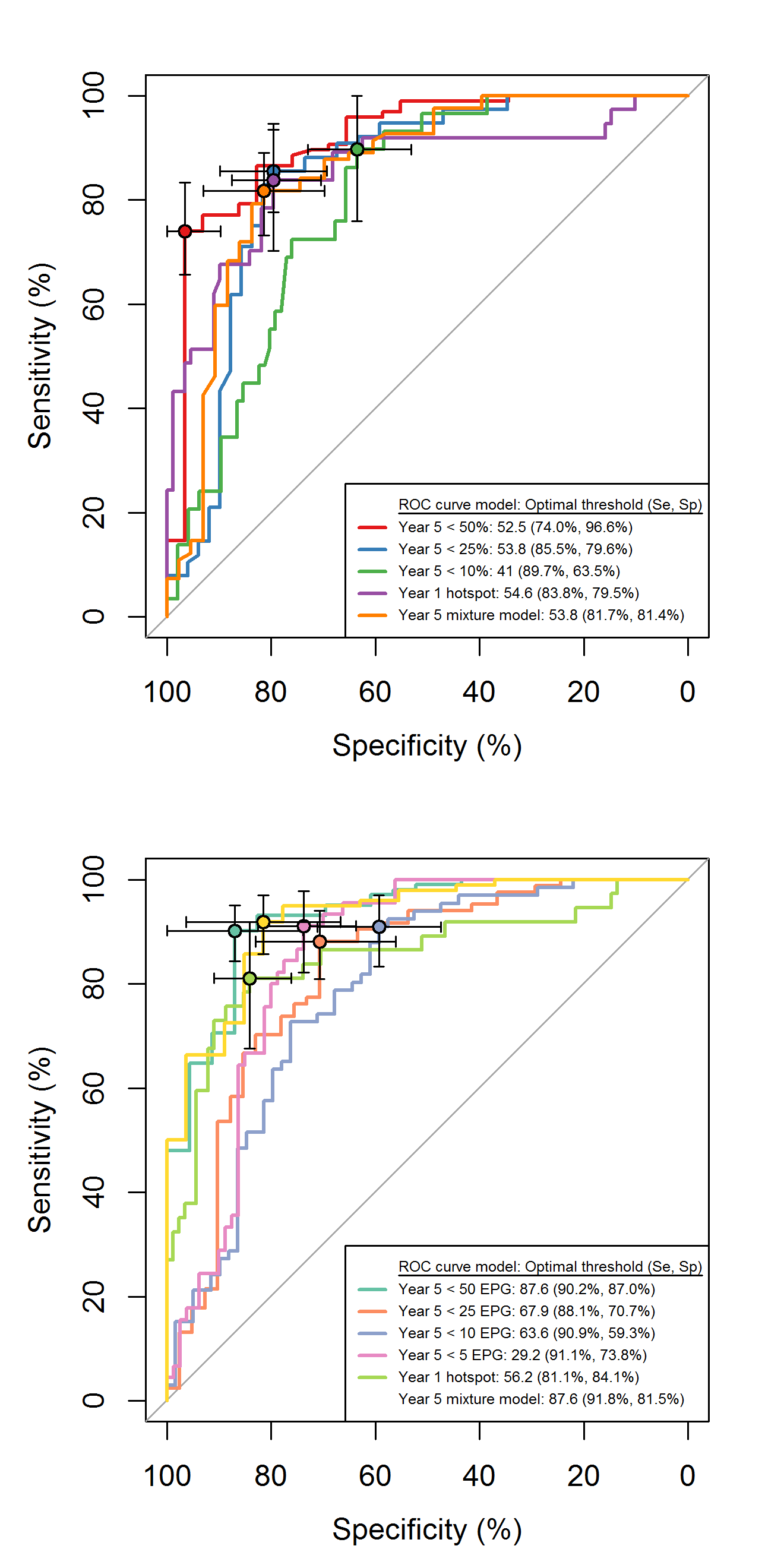


### **Figure S15**. ROC curves for determination of optimal cutoff at year 3 for prevalence (top panel) and mean intensity (bottom panel) using multiple thresholds.


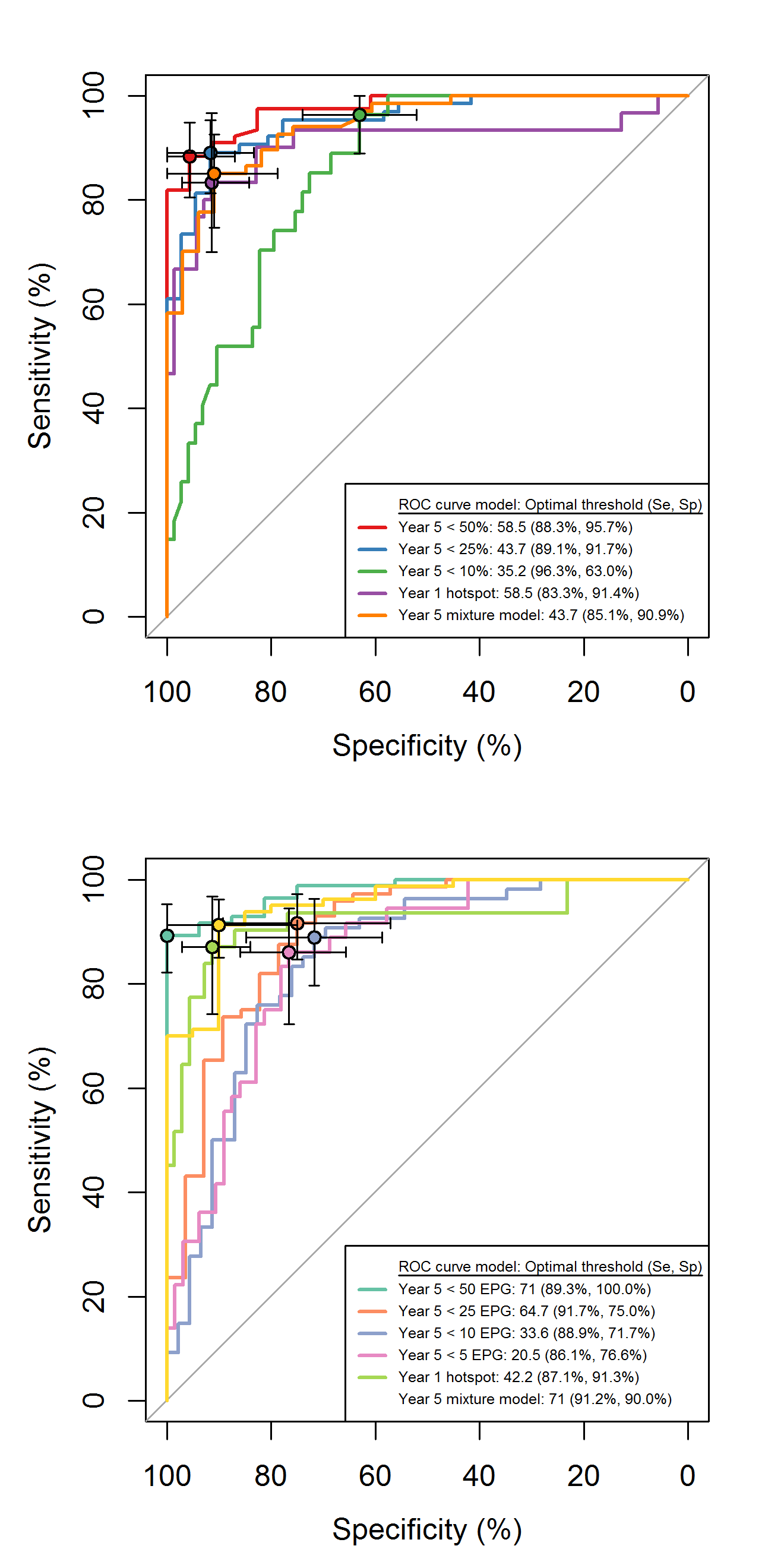


### **Figure S16**. ROC curves for determination of optimal cutoff at year 4 for prevalence (top panel) and mean intensity (bottom panel) using multiple thresholds.


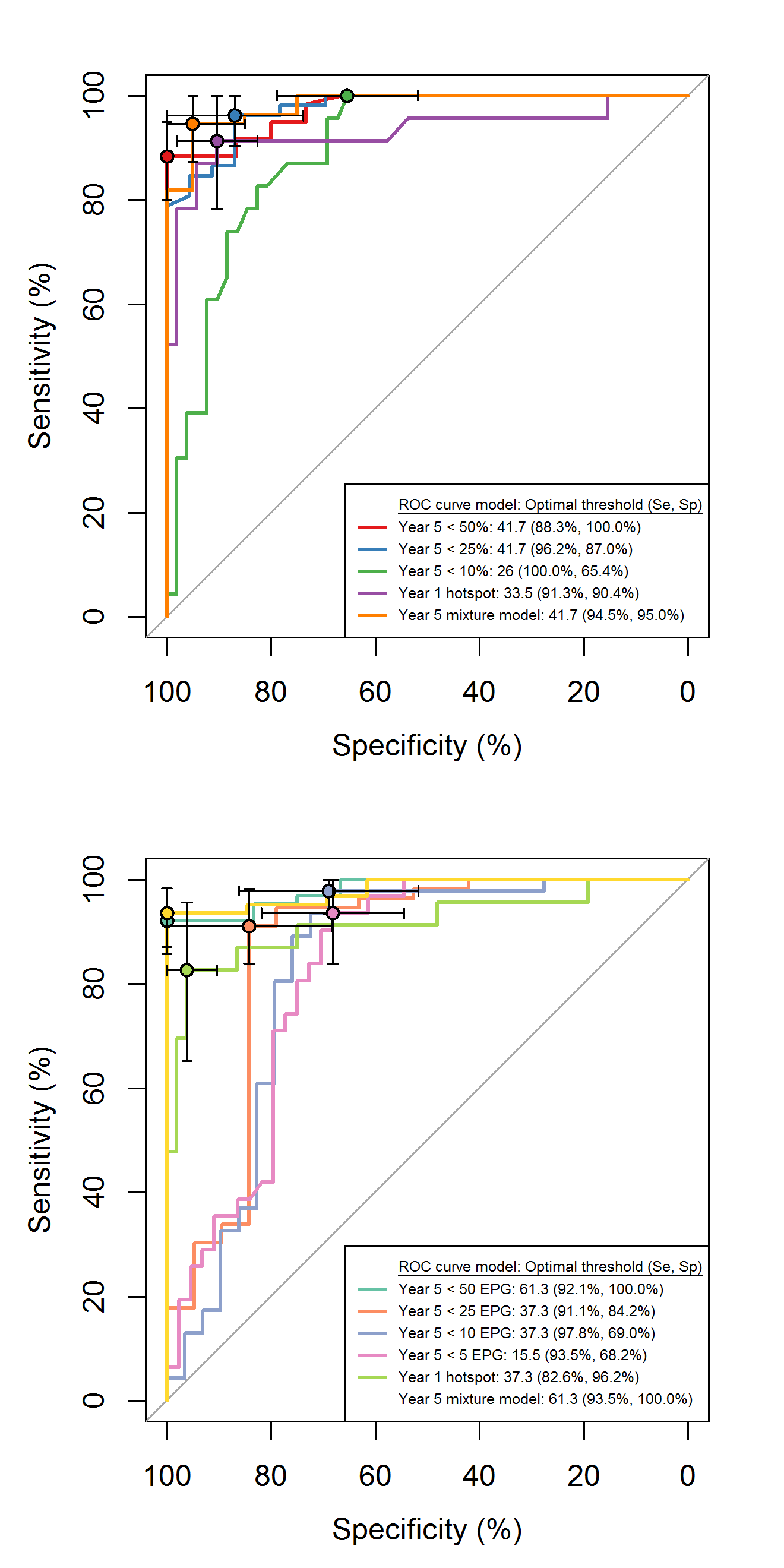


### **Figure S17**. ROC curves for determination of optimal cutoff at year 5 for prevalence (top panel) and mean intensity (bottom panel) using multiple thresholds.


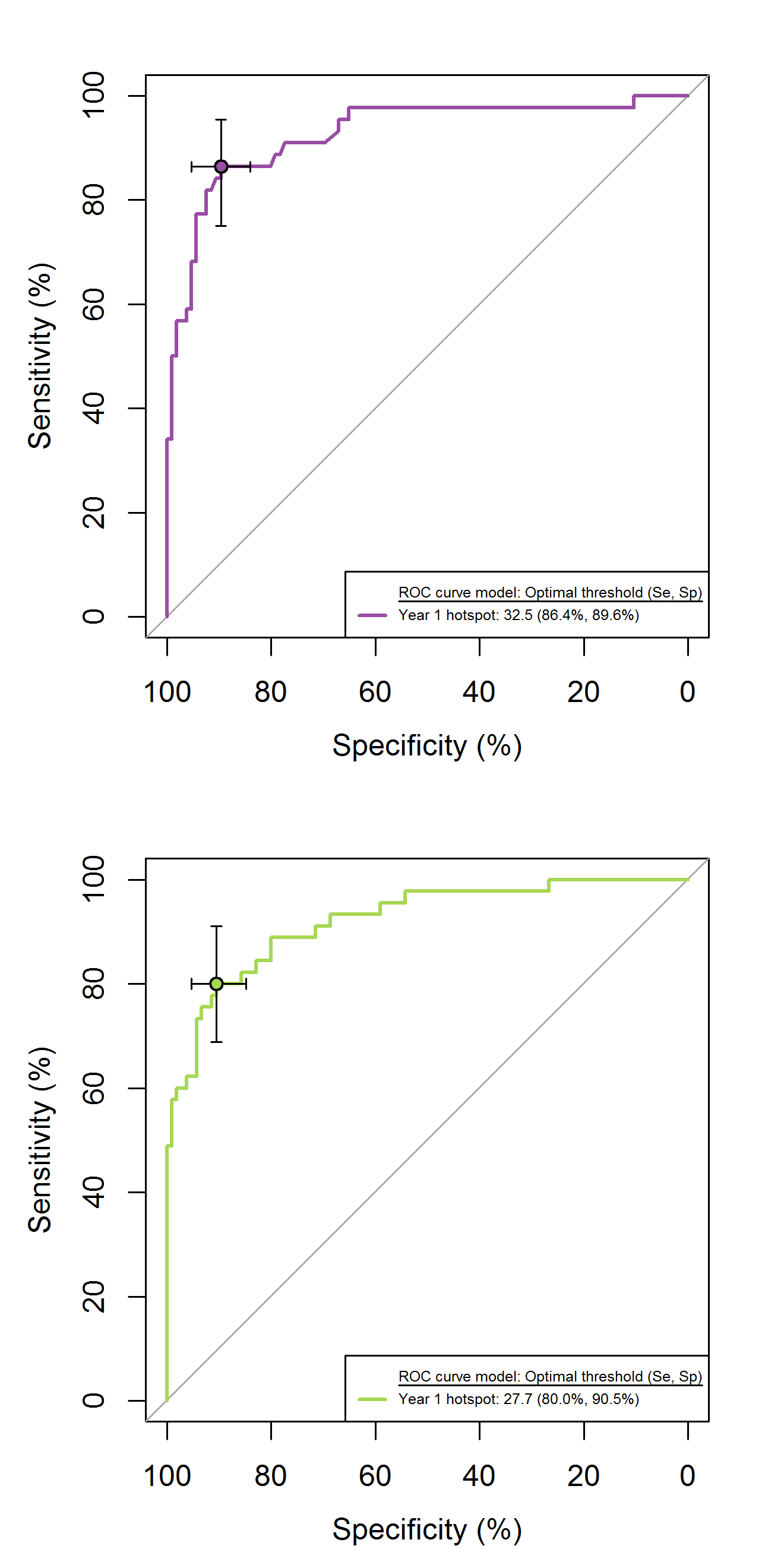


### **Figure S18**. ROC curves for determination of optimal cutoff using the mean of years 1 and 2 for prevalence (top panel) and mean intensity (bottom panel) using multiple thresholds.


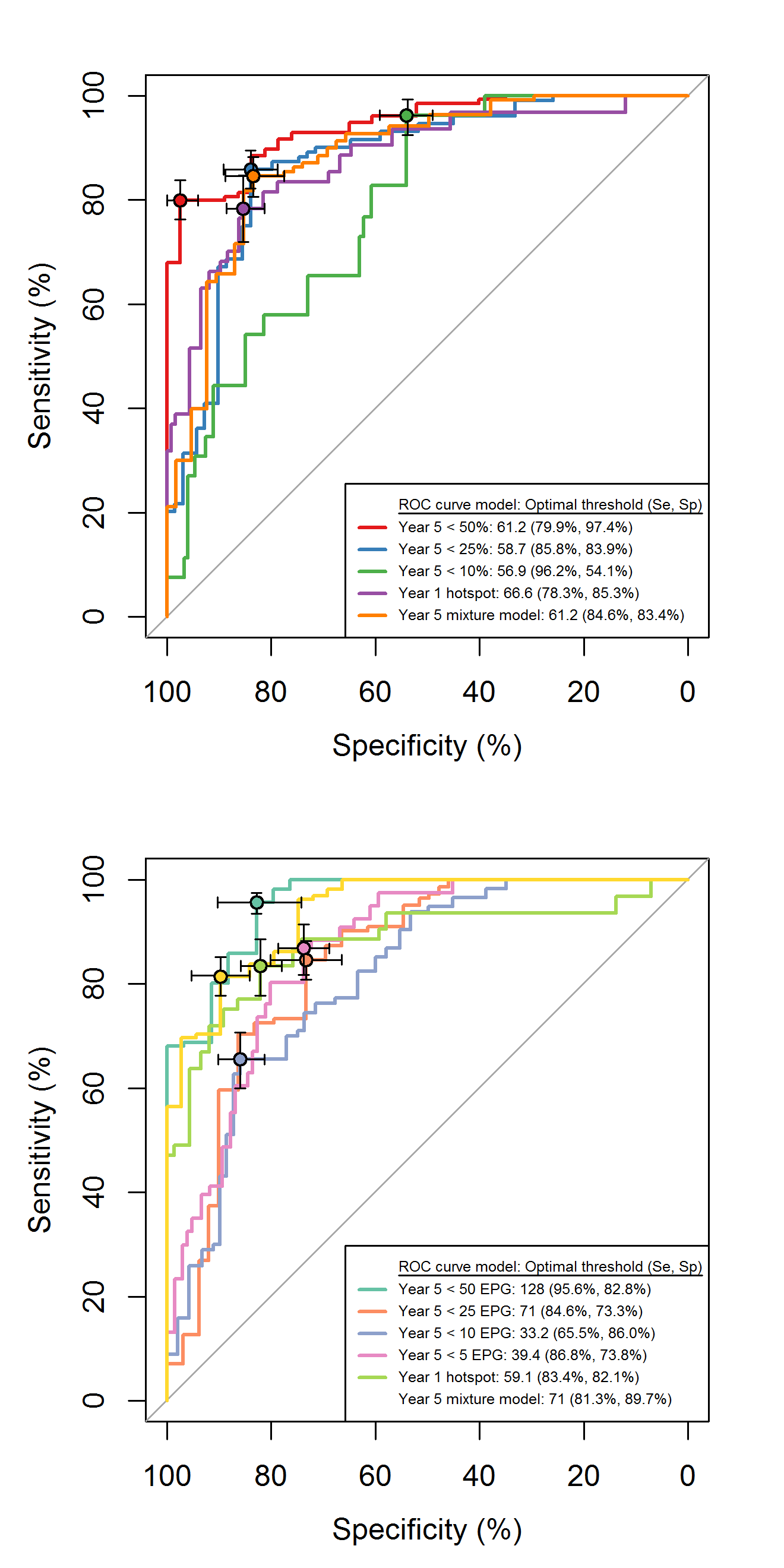


### **Figure S19**. ROC curves for determination of optimal cutoff using the mean of years 1, 2, and 3 for prevalence (top panel) and mean intensity (bottom panel) using multiple thresholds.


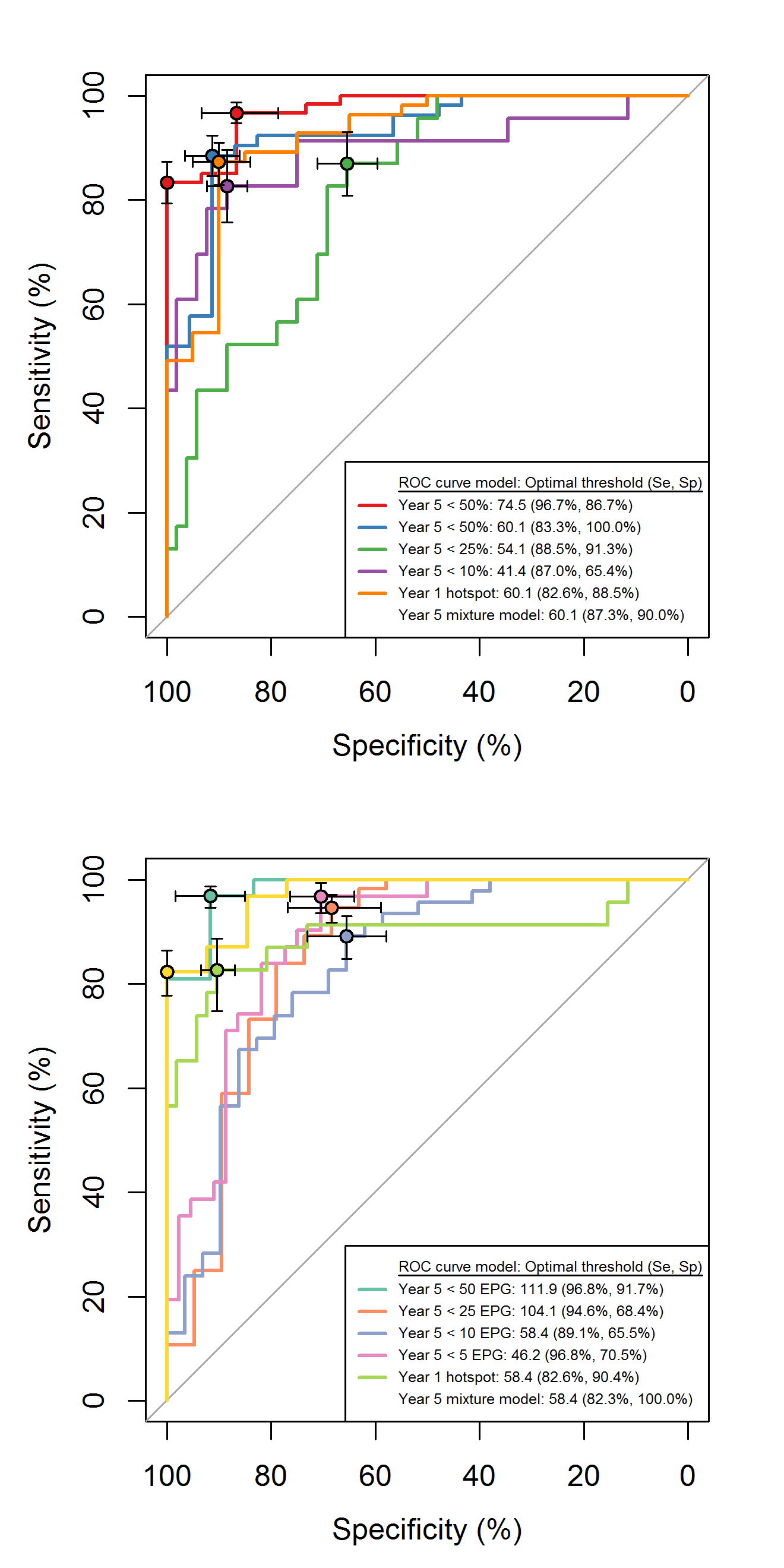


### **Figure S20**. ROC curves for determination of optimal cutoff using the change at year 2 from year 1 for prevalence (top panel) and mean intensity (bottom panel) using multiple thresholds.


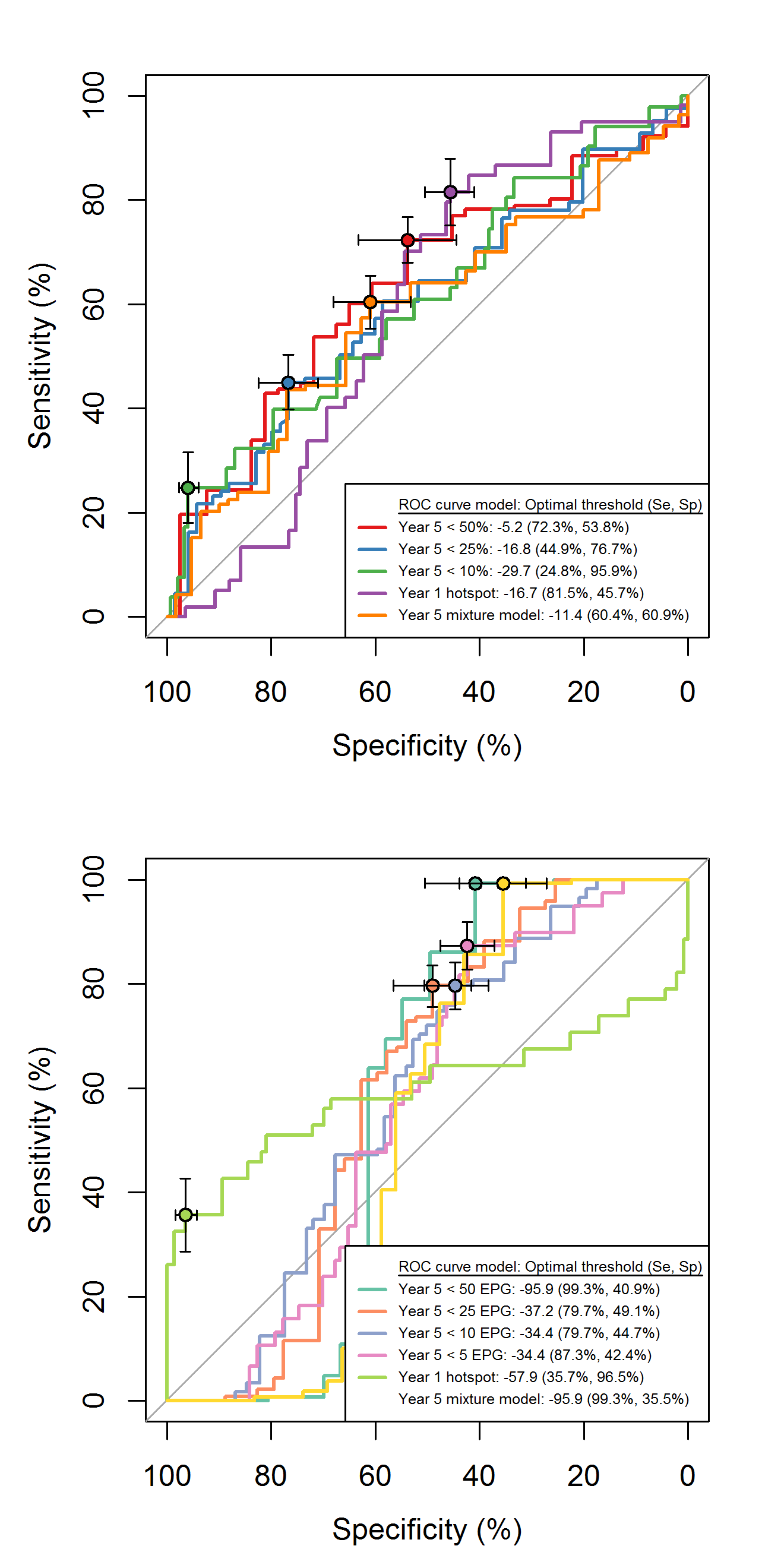


### **Figure S21**. ROC curves for determination of optimal cutoff using the change at year 3 from year 1 for prevalence (top panel) and mean intensity (bottom panel) using multiple thresholds.


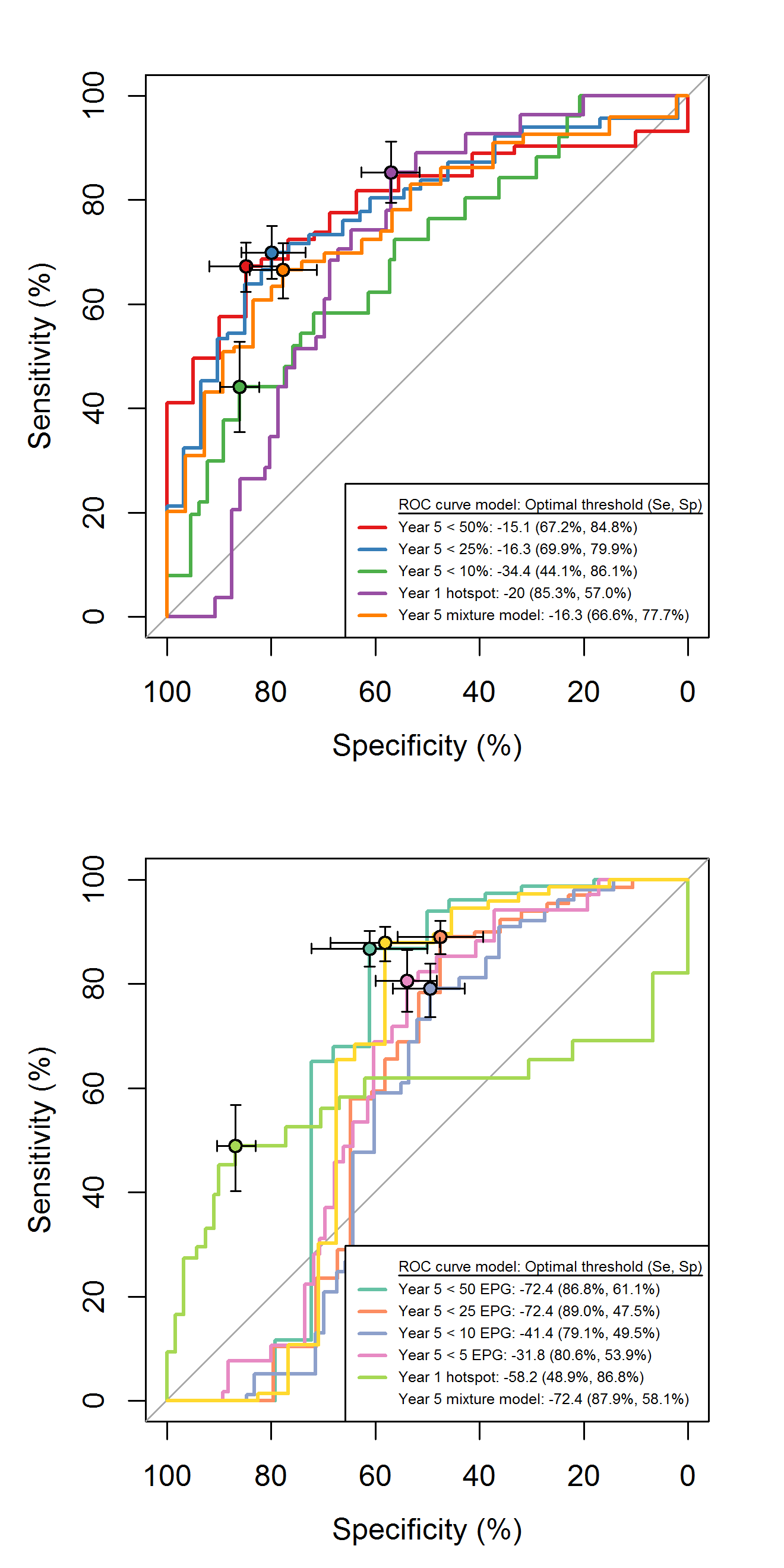

Supplement: Supplementary Material [file jix496_suppl_supplementary_material.docx]
